# Application of Gene Expression Trajectories Initiated from ErbB Receptor Activation Highlights the Dynamics of Divergent Promoter Usage

**DOI:** 10.1371/journal.pone.0144176

**Published:** 2015-12-14

**Authors:** Daniel Carbajo, Shigeyuki Magi, Masayoshi Itoh, Hideya Kawaji, Timo Lassmann, Erik Arner, Alistair R. R. Forrest, Piero Carninci, Yoshihide Hayashizaki, Carsten O. Daub, FANTOM consortium, Mariko Okada-Hatakeyama, Jessica C. Mar

**Affiliations:** 1 Department of Systems and Computational Biology, Albert Einstein College of Medicine, Bronx, NY, United States of America; 2 Laboratory for Integrated Cellular Systems, RIKEN Center for Integrative Medical Sciences, Tsurumi-ku, Yokohama, Japan; 3 RIKEN Center for Life Science Technologies (Division of Genomic Technologies), Tsurumi-ku, Yokohama, Japan; 4 RIKEN Omics Science Center, Tsurumi-ku, Yokohama, Japan; 5 RIKEN Preventive Medicine and Diagnosis Innovation Program, Wako-shi, Japan; 6 Telethon Kids Institute, The University of Western Australia, Subiaco, Western Australia, Australia; 7 Department of Medicine, Karolinska Institutet and Center for Metabolism and Endocrinology, Karolinska University Hospital, Stockholm, Sweden; 8 Harry Perkins Institute of Medical Research, QEII Medical Centre and Centre for Medical Research, The University of Western Australia, Nedlands, Western Australia, Australia; 9 Department of Biosciences and Nutrition and Science for Life Laboratory, Karolinska Institutet, Stockholm, Sweden; 10 Department of Epidemiology and Population Health, Albert Einstein College of Medicine, Bronx, NY, United States of America; Medical College of Wisconsin, UNITED STATES

## Abstract

Understanding how cells use complex transcriptional programs to alter their fate in response to specific stimuli is an important question in biology. For the MCF-7 human breast cancer cell line, we applied gene expression trajectory models to identify the genes involved in driving cell fate transitions. We modified trajectory models to account for the scenario where cells were exposed to different stimuli, in this case epidermal growth factor and heregulin, to arrive at different cell fates, i.e. proliferation and differentiation respectively. Using genome-wide CAGE time series data collected from the FANTOM5 consortium, we identified the sets of promoters that were involved in the transition of MCF-7 cells to their specific fates versus those with expression changes that were generic to both stimuli. Of the 1,552 promoters identified, 1,091 had stimulus-specific expression while 461 promoters had generic expression profiles over the time course surveyed. Many of these stimulus-specific promoters mapped to key regulators of the ERK (extracellular signal-regulated kinases) signaling pathway such as FHL2 (four and a half LIM domains 2). We observed that in general, generic promoters peaked in their expression early on in the time course, while stimulus-specific promoters tended to show activation of their expression at a later stage. The genes that mapped to stimulus-specific promoters were enriched for pathways that control focal adhesion, p53 signaling and MAPK signaling while generic promoters were enriched for cell death, transcription and the cell cycle. We identified 162 genes that were controlled by an alternative promoter during the time course where a subset of 37 genes had separate promoters that were classified as stimulus-specific and generic. The results of our study highlighted the degree of complexity involved in regulating a cell fate transition where multiple promoters mapping to the same gene can demonstrate quite divergent expression profiles.

## Introduction

Cell fate transitions occur via the tight, temporal coordination between signaling pathways used by a cell. While the makeup and control of these pathways are highly specific to the stimulus used to induce the transition, a common theme that these pathways share is the activation of cell surface receptors that trigger the initial early response of signaling cascades that then lead to the expression of genes that facilitate the transition. Defining the input and output components of the signaling cascade is feasible, however identifying the transcriptional programs that bridge these two endpoints remains somewhat of a black box. Understanding how cells are regulated by key genes and their corresponding networks during fate transitions may also present opportunities to restrict or manipulate cells towards specific endpoints, an application that has utility in the development of new cancer therapies.

Elucidation of the genes expressed during fate transitions represents a critical component to understanding how cells use signaling pathways to change their fate. High-throughput technologies have improved our ability to refine the list of signaling components that contribute to a cell fate transition. However even with access to the best technology available, our limitation in understanding signaling is constrained more by the fact that pathways operate as complex, non-linear circuits, and that their usage by cells to achieve transitions is far more complicated than a simple input-output system [1, 2].

Despite the complex arrangement of signaling components that underlie cell fate transitions, in nature, there is clearly convergence of only a finite number of possible pathways that are used by the cell. This phenomenon is most readily understood when we consider known cases where different stimuli can trigger cells to adopt the same phenotypic outcome. For example, human HL-60 promyelocytic cells when exposed to dimethyl sulfoxide (DMSO) and all-trans retinoic acid (ATRA) both lead to neutrophil differentiation [3, 4]. Examples also exists where different stimuli lead to distinct outcomes, e.g. for the PC-12 cell line, stimulation of the cells by nerve growth factor (NGF) induces differentiation, while stimulation by epidermal growth factor (EGF) induces proliferation [5]. Another example is the ErbB receptor signaling pathway, where exposure of MCF-7 cells to EGF results in proliferation, whereas exposure to heregulin (HRG) leads to differentiation [6, 7]. In those cell systems, EGF induces transient activation of ERK (extracellular signal-regulated kinases) whereas NGF and HRG induce sustained activation of ERK, of which the duration is thought to be critical to cell fate determination, and therefore it may induce different gene expression trajectories.

Both EGF and HRG ligands share a common ErbB receptor signaling pathway. Upon activation of the ErbB receptors, a multi-layered signal transduction network is initiated, often involving the activation of the ERK and the phosphatidiylinositol 3'-kinase (PI3K) pathways [8, 9]. EGF and HRG produce qualitatively similar immediate early responsive gene (IEG) profiles of the genes involved in the ERK pathway, though there are large quantitative differences. This suggests that quantitative changes in the expression levels of IEGs, and not the predominant regulation of a few specific ones, would be followed by robust qualitative differences in successive gene expression waves, eventually leading to the different cell fates observed that reflect ligand specificity [6, 7, 10]. This observation points to the value of carefully considering the relative changes in expression during the transition as a means to identify fate-specific regulators.

Gene expression trajectory models were initially developed to identify the two major sets of genes involved in a cell fate transition [11]. One set involves those genes driving core signaling pathways that lead to a common endpoint, and a second set of genes that have divergent expression profiles that are specific to the stimulus applied. The focus of our current study was to adapt trajectory models for time courses where different stimuli cause transitions to distinct fates. The application of trajectory models to MCF-7 cells exposed to EGF and HRG represents an opportunity to elucidate genes that are cell fate-specific from those that are expressed in a cell fate-generic manner.

Cap analysis of gene expression (CAGE) is a technique that was pioneered to identify the location of transcription start sites (TSSs) in the genome and quantify their usage [12]. Two CAGE time course data sets were collected for MCF-7 cells exposed to EGF and HRG separately for fifteen time points spanning 0hr (non-treated) to 8hr, with three biological replicates (see Materials and Methods). These data sets were part of the FANTOM5 time courses, which comprehensively showed coordinated transcriptional waves in transitioning mammalian cells [13]. Using CAGE, we are able to obtain a more accurate snapshot of transcriptional regulation by profiling at the resolution of individual promoters than using other high-throughput methods like RNA-seq or microarrays.

Alternative promoters are defined as multiple promoter sites in the genome that control the same gene [14], and confer robustness in the genome by ensuring that production of a transcript will occur by having multiple TSSs available. By adapting the trajectory models to be applicable for usage with promoter-level data obtained from CAGE, we have added insight to how cell fate transitions are being regulated at a deeper layer of signaling complexity by identifying alternative promoter usage in the MCF-7 time courses. This work is part of the FANTOM5 Phase 2 project. Data download, genomic tools and co-published manuscripts have been summarized at http://fantom.gsc.riken.jp/5/.

## Materials and Methods

### Data set

The MCF-7 human breast cancer cell line was exposed to either EGF or HRG, and samples were collected at 0h (non-treated), 15min, 30min, 45min, 60min, 80min, 100min, 2hr, 2.5hr, 3hr, 3.5hr, 4hr, 5hr, 6hr, 7hr and 8hr. These time points were selected in order to sample the early phase of cell differentiation as well as capture mid and delayed-stages of gene expression. The data was profiled using HeliscopeCAGE, resulting in time-course expression levels of 102,540 promoters under the two treatments (HRG and EGF). The time courses each had three biological replicates for each time point at all 15 time points (including the time 0, non-treated sample).

### Filtering steps

Promoters with expression of less than 1 tag per million for all time points were removed, resulting in a total of 54,822 promoters. Using log_2_-transformed data, we fitted an ANOVA model of the form:

$$Y_{ik}=\alpha_{0}+time_{i}+\varepsilon_{ik}$$

to each promoter for the EGF and HRG time courses separately, where *i* denotes the time point indices from 1 to 15, and *k* denotes the three biological replicates. A promoter was removed from the analysis if it had no statistically significant change in expression over time for both treatments (adjusted P-value > 0.01). The P-values from the ANOVA model were adjusted for multiple testing using the Benjamini-Hochberg method [15]. A total of 1,552 promoters were retained from this step.

Identifying generic and stimulus-specific promoters

An ANOVA model was constructed for each individual promoter:

$$Y_{ijk}=\beta_{0}+time_{i}+stimulus_{j}+\varepsilon_{ijk}$$

A promoter that was statistically significant for the *stimulus* term (adjusted P-value < 0.01) was assigned to the fate-specific set of promoters, whereas a promoter that failed to attain significance was classified as generic. P-values were adjusted using the Benjamini-Hochberg method [15] for multiple testing correction.

### Detecting divergence in promoter expression over time

Fold change of the expression profile of HRG:EGF treatment was calculated for each promoter. An ANOVA model was fitted to detect which promoters had significant fold change differences across time (adjusted P-values < 0.01). P-values were corrected for multiple testing using the Benjamini-Hochberg method.

### Promoter to gene mapping

Promoters were mapped to their respective gene-centric annotations using the FANTOM5 expression atlas [16] and HGNC-EntrezGene relationships downloaded from http://www.genenames.org/cgi-bin/hgnc_downloads.cgi. In most cases, there was a one-to-one relationship between a promoter and a gene. 319 out of the 461 generic promoters mapped to 306 genes with known EntrezGene IDs and 847 out of the 1,091 stimulus-specific promoters mapped to 737 genes with known EntrezGene IDs.

### Identifying transcription factors

Transcription factor sets were defined from the TFcheckpoint database [17] which represents a comprehensive repository of putative transcription factors according to experimental evidence based on their function as true sequence-specific DNA-binding RNA polymerase II regulators. 100 transcription factors were identified among the 1,006 different genes, 38 transcription factors mapped to generic promoters and 68 transcription factors were mapped to stimulus-specific promoters (6 of them shared, as controlled by promoters classified in both groups).

### Functional enrichment analyses

We wrote R code to test for over-representation of Gene Ontology (GO) terms and KEGG pathways in the genes that were mapped by the stimulus-specific and generic promoter sets. Significance of enrichment was assessed using the Fisher’s exact test. Annotations were based on GO and KEGG terms as defined by the Bioconductor packages org.Hs.eg.db (version 2.10.1) and KEGG.db (version 2.10.1). P-values were adjusted for multiple testing using the Benjamini-Hochberg method, using a significance threshold of 0.05. Only genes with evidence codes based on experimental validation in GO were used for this analysis, specifically evidence codes EXP (inferred from experiment), IDA (inferred from direct assay), IPI (inferred from physical interaction), IMP (inferred from mutant phenotype) or IGI (inferred from genetic interaction). We also discarded child nodes of GO terms to improve the specificity of the results obtained. We compared our results to the DAVID (Database for Annotation, Visualization and Integrated Discovery, version 6.7) web interface [18] as well as the GOstats Bioconductor package (version 2.31.0) and observed similar results to those obtained with our approach. Our R functions are freely available upon request.

### Differential co-expression analysis

Expression profiles of the promoters mapping to the same gene were averaged to create a single expression profile for each unique gene. Changes in gene expression correlation in the two EGF and HRG time courses were explored using an empirical Bayesian approach for identifying differentially co-expressed gene pairs implemented in the Bioconductor EBcoexpress package [19] (version 1.10.0). Co-expression networks were visualized using the R package igraph (version 0.7.1).

### Protein-protein interaction networks reconstruction

Sets of generic and stimulus-specific transcription factor interactions were derived using the *shortest*.*paths* tools from the R package igraph (version 0.7.1), and were applied to a protein-protein interaction (PPI) network constructed from iRefIndex [20] data. The PPI network was reconstructed using the R package iRefR (version 1.13) [21] which bridges the iRefIndex database (version 13.0) to an R environment. This database consolidates PPI data available in multiple primary interaction databases, namely BIND [22], BioGRID [23], CORUM [24], DIP [25], HPRD [26], IntAct [27], MINT [28], Mpact [29], MPPI [30], and OPHID [31] in a non-redundant fashion. A minimal sub-network connecting the 38 transcription factors controlled by generic promoters (32 controlled by generic promoters exclusively, and 6 controlled by alternative promoters classified as either generic or stimulus-specific) was extracted from the network as a sub-network of shortest paths between them. The same procedure was used to reconstruct an analogous sub-network of shortest paths between the 68 transcription factors controlled by stimulus-specific promoters (62 controlled by stimulus-specific promoters exclusively, and 6 controlled by alternative promoters classified as either generic or stimulus-specific).

### Comparison of results from the CAGE data set with other gene expression data sets

The microarray and qPCR data sets were obtained from the authors of [10]. For the microarray data set, the same analysis as what was applied to the CAGE data set was used to identify the generic and stimulus-specific genes. For the qPCR data set, the design of this time course was sparser and therefore enough data was not available to fit the same models directly as in the case of the microarray data. Instead, we used the Pearson correlation coefficient, *R*, as the statistic to determine whether a gene was generic (*R* > 0.5) or stimulus-specific (*R* ≤ 0.5) in the qPCR data set. A two-sided Fisher’s exact test was used to assess the significance of overlap observed between the CAGE data sets and the two other gene expression data sets. See S1 Text for more details.

## Results

### Application of trajectory models to classify promoters into generic and stimulus-specific sets identifies the differential nature of transcriptional regulation underlying the cell fate transition

**Fig 1:**
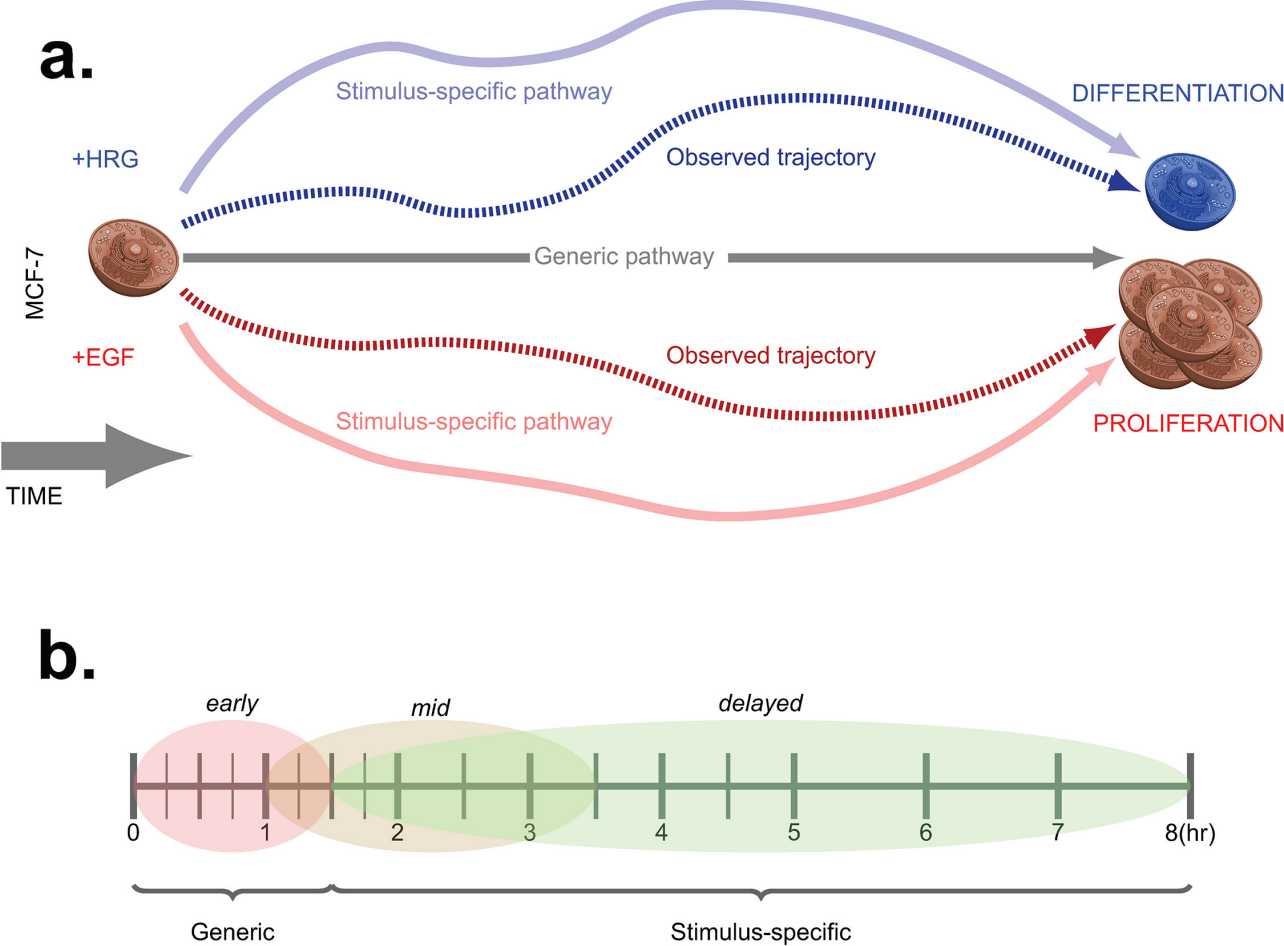
Outline of the trajectory models and experimental design of the data. **A.** Schematic view of the gene expression trajectory models for MCF-7 cells undergoing proliferation or differentiation in response to EGF or HRG, respectively. **B.** Experimental design of the time course experiments where time points were selected to cover early to late stages of the cell fate transition.

The first step to understanding how cells use transcriptional programs to transition to different endpoints is to identify the genes whose coordinated expression during the transition is specific to one cell fate versus another. In comparing the time course data for EGF and HRG-stimulated MCF-7 cells, the subsets of promoters that display either generic expression, where profiles are preserved across stimuli, or stimulus-specific expression profiles (see Fig 1A) can be identified by applying the following ANOVA model to each individual promoter:

$$Y_{ijk}=\beta_{0}+time_{i}+stimulus_{j}+\varepsilon_{ijk}$$ (1)

where *i* corresponds to each time point in the experiment and ranged from 1 to 15, *j* represents the stimulus applied (either EGF or HRG), and *k* denotes each of the three biological replicates used in the time course data (see Fig 1B).

A promoter was classified into the stimulus-specific group if the *stimulus* variable in the ANOVA model was statistically significant (adjusted P-value < 0.01) since this corresponds to a scenario where the expression profiles deviate substantially between the EGF and HRG-stimulated time courses. A promoter failing to attain statistical significance for the *stimulus* variable was classified in the generic group (adjusted P-value > 0.01), which signifies coordination of the expression profiles between the two stimuli. An initial filtering step was applied to all promoters to exclude those with no significant change across both times courses as these promoters were unlikely to be informative for understanding the transcriptional program underlying the cell fate transition (see Materials and Methods).

**Fig 2:**
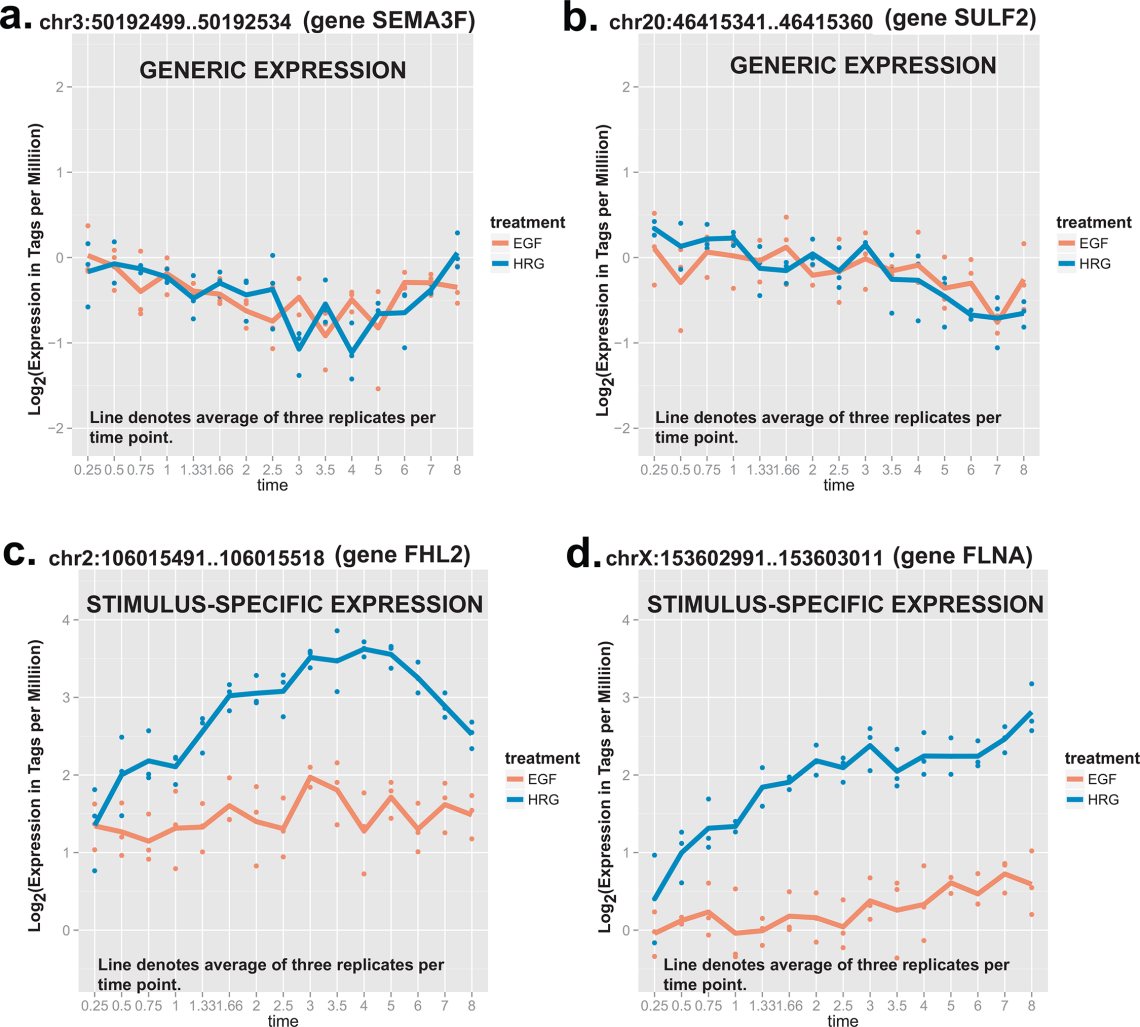
Examples of promoters with generic and stimuli-specific expression profiles. **A.** the promoter region maps to gene SEMA3F and has a generic expression profile across the EGF and HRG profiles. **B.** the promoter mapping to SULF2 also has a generic profile. **C.** the stimuli-specific promoter maps to gene FHL2 and **D.** the stimuli-specific promoter maps to gene FLNA.

**Fig 3:**
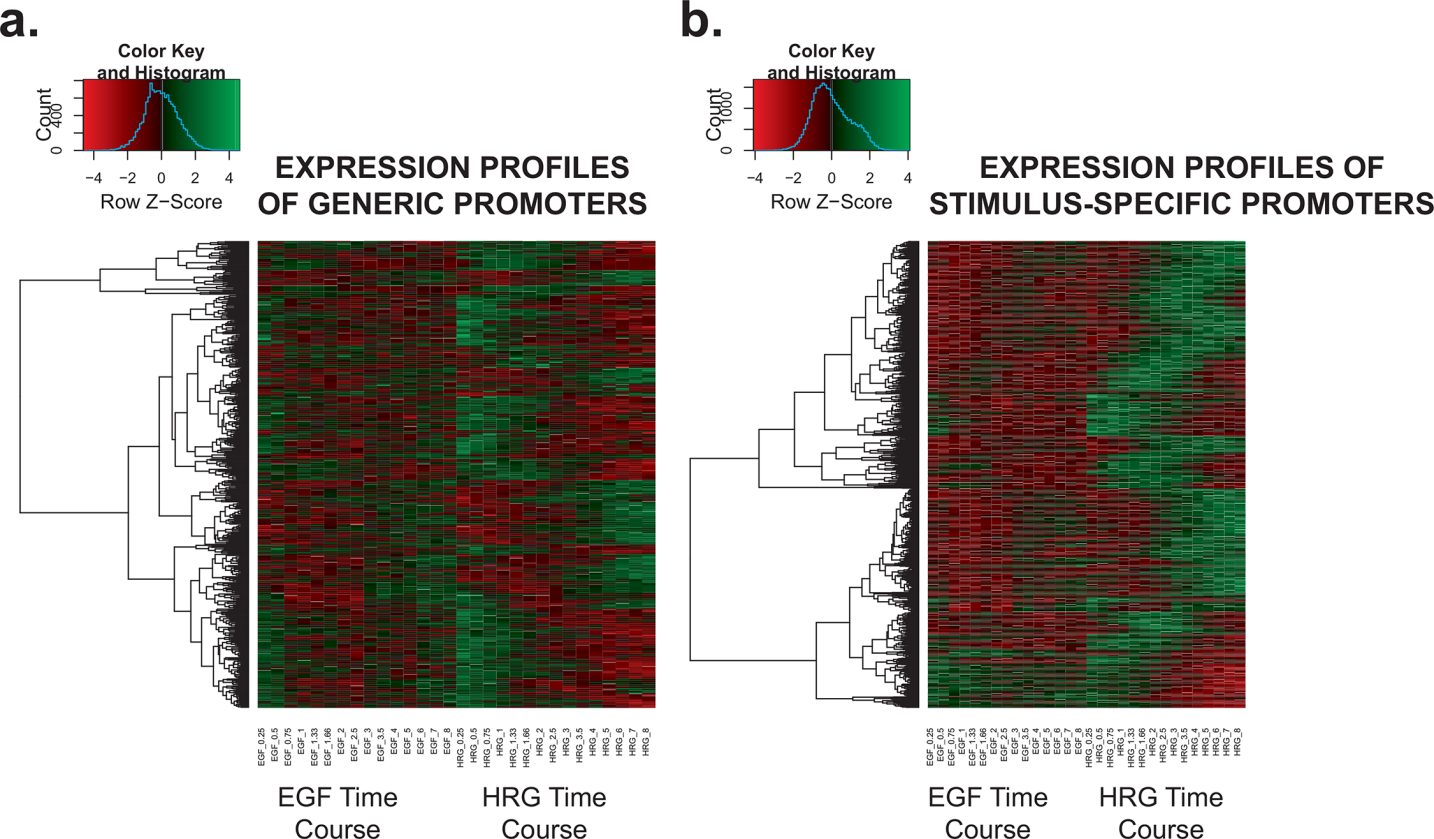
Expression activity of the generic and stimulus-specific promoters across the time course. Heat maps showing the expression levels of promoters in the **A.** generic and **B.** stimulus-specific groups under EGF or HRG treatments. Expression levels have been scaled by row (promoter). Generic promoters drive predominantly immediate early expression, while stimulus-specific ones drive mid-delayed expression; expression changes are mainly driven by the HRG treatment, being much less obvious and dramatic after EGF stimulation.

Based on our classification scheme, we obtained 461 generic and 1,091 stimulus-specific promoters (representing 30% and 70% of all promoters tested, respectively). Examples of promoters with generic and stimulus-specific expression profiles are shown in Fig 2A and 2D. Heatmaps also demonstrate how expression changes are occurring for the most highly-expressed subset of generic and stimulus-specific promoters in the two time courses (see S1A and S1B Fig). Amongst the list of promoters identified, we observed some promoters control genes with an established role in determining specific cell fate decisions. The generic promoters are associated with early expression that decreases over time and have a role in regulating cell signaling, for example, STRAP (serine/threonine kinase receptor associated protein) [32], ULK1 (unc-51 like autophagy activating kinase 1) [33], and SEMA3F (semaphorin 3F, see Fig 2A) [34]. One the other hand, one of the most significant stimulus-specific promoters drives the transcription of an important regulator, FHL2 (four and a half LIM domains 2) (see Fig 2C), which can switch off upstream ERK signals and also binds to FOS (Finkel–Biskis–Jinkins murine osteogenic sarcoma virus) and/or FRA1 (FOS-related antigen 1) (AP-1 family members proteins [35]. Another significantly stimulus-specific gene FLNA (filamin A) (see Fig 2D), the actin-binding and scaffolding protein, positively regulates ERK activation and cell shape change together with β-arrestins [36]. These stimulus-specific genes cooperatively act to fine-tune ERK activity, which is critical for signal-dependent cell fate control [2, 9]. From our results we note that the majority of generically-regulated promoters are activated at early time points and their expression levels tend to decrease over time, whereas many of the stimulus-specific promoters are activated at delayed time points and their expression tends to increase over time (Fig 3A and 3B), indicating the divergence of cellular state along the time course.

### Trajectory models highlight the differences occurring in the gene expression dynamics of generic and stimulus-specific promoters for the EGF and HRG-stimulated MCF-7 cells

The definitions of generic and stimulus-specific promoters were used as a foundation to further investigate the expression dynamics of genes involved in the cell fate transition. A related but simpler model than (1) that can be fit to each individual promoter is a linear regression model that is specified as follows:

$$Y_{ijk}=\alpha_{0}+\alpha \cdot time_{i}+\lambda \cdot stimulus_{j}+\varepsilon_{ijk}$$ (2)

where *α* is the regression coefficient that measures the direction and degree of impact that an increase in time will have on the expression of a specific promoter. We can capitalize on this property and use the direction of the estimated *α* from the model in (2) to infer whether promoters were associated with an overall increase (*α* > 0) or decrease (*α* < 0) in expression during the time course. The sets of generic and stimulus-specific promoters were subset further based on the direction of the estimated *α* (see Table 1). In the generic promoter group, we detected far more promoters with decreasing expression (289 promoters) compared to those whose expression increased over the time course (172 promoters). Further inspection of the generic promoters revealed that activation of the majority of the generic promoters occurred early on in the time course (see Fig 3A). Activation of the stimulus-specific promoters on the other hand showed greater divergence between the EGF and HRG time courses (see Fig 3B). In the EGF-exposed cells the stimulus-specific promoters followed a pattern of divergence in expression, whereas this was in contrast to the HRG-exposed cells that showed these promoters experienced a marked shift in expression over time. This shift was notably absent from the EGF time course data. A greater number of stimulus-specific promoters showed increasing expression over the time course (628 promoters) as compared to those whose expression decreased (463 promoters).

**Table 1:** The top ten most significant generic and stimulus-specific promoters.

Status of Promoter Expression	Promoter	Gene Symbol	Adjusted P-value	Regression Coefficient from the Linear Model
Generic	*chr2*:*238395772*..*238395830*,*-*	*NA*	0.995	-0.03
	*chr3*:*124732396*..*124732408*,*-*	HEG1	0.989	-0.01
	*chr12*:*132379245*..*132379256*,*+*	ULK1	0.985	-0.1
	*chr20*:*46415341*..*46415360*,*-*	SULF2	0.985	-0.05
	*chr10*:*99185961*..*99185987*,*+*	PGAM1	0.969	0.03
	*chr16*:*67515264*..*67515316*,*+*	*NA*	0.964	0.02
	*chr19*:*48972612*..*48972632*,*+*	CYTH2	0.962	-0.03
	*chr9*:*34665595*..*34665628*,*+*	*NA*	0.961	-0.06
	*chr3*:*50192499*..*50192534*,*+*	SEMA3F	0.960	-0.03
	*chr12*:*16035307*..*16035352*,*+*	STRAP	0.960	0.02
Stimulus-specific	*chr10*:*54074033*..*54074050*,*+*	DKK1	6.38E-022	0.04
	*chr6*:*43737939*..*43737956*,*+*	VEGFA	6.23E-022	0.05
	*chr2*:*106015491*..*106015518*,*-*	FHL2	5.55E-022	0.06
	*chr14*:*69445991*..*69446029*,*-*	ACTN1	4.91E-022	0.04
	*chr7*:*143078379*..*143078454*,*+*	ZYX	2.78E-022	0.09
	*chr1*:*86046433*..*86046453*,*+*	CYR61	7.29E-024	-0.05
	*chr21*:*40177845*..*40177863*,*+*	ETS2 (TF)	2.65E-024	-0.02
	*chr3*:*5021113*..*5021180*,*+*	BHLHE40 (TF)	2.65E-024	0.03
	*chrX*:*153602991*..*153603011*,*-*	FLNA	2.15E-029	0.09
	*chr10*:*75757863*..*75757897*,*+*	VCL	7.46E-030	-0.01

Promoters that map to known transcription factors are denoted with TF.

### Key stimulus-specific promoters have significantly divergent profiles during the cell fate transition

**Fig 4:**
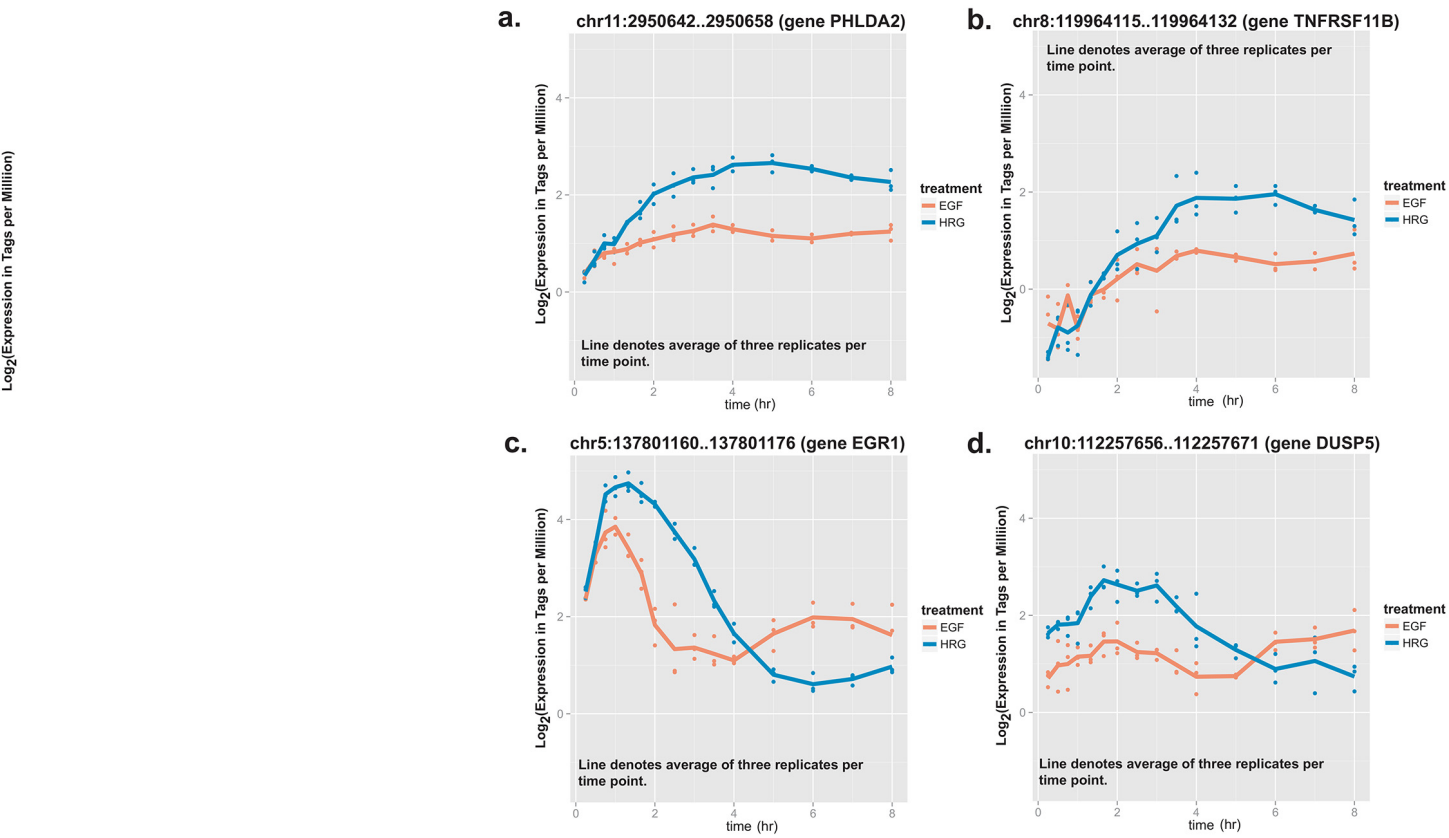
Time-course expression profiles of stimulus-specific promoters associated with significant fold changes of HRG-induced expression versus EGF-induced expression. **A.** and **B.** show examples of promoters with an overall increase in expression. **C.** and **D.** show promoters with an overall decrease in expression.

The stimulus-specific promoters identified by trajectory models represent promoters with divergent expression profiles during the MCF-7 time course. ItIt is possible that the size of the divergence in expression may be correlated with the level of importance a gene plays in regulating the cell fate transition. Within the subset of stimulus-specific promoters, we looked for promoters with increasingly larger divergence in expression between the two stimuli during the time course. Seven stimulus-specific promoters (see Table 2) were identified with significant increments in expression at successive time points in the HRG-stimulated time course versus the EGF one (see Fig 4A–4D, Materials and Methods). These promoters control the expression of six unique genes KRT15 (keratin 15), TNFRSF11B (tumor necrosis factor receptor superfamily member 11b), TMEM185B (transmembrane Protein 185B), PHLDA2 (pleckstrin homology-like domain, family A member 2), DUSP5 (dual specificity phosphatase 5), EGR1 (early growth response 1) (see Table 2). Two promoters are associated with an overall decrease in gene expression for two genes previously mentioned (EGR1 and DUSP5). These two promoters drive higher expression levels in the EGF-stimulated time course compared to the HRG-stimulated time course at later time points… For both promoters, there is a cross-over point where expression in the EGF-exposed cells is higher than the HRG-exposed cells, suggesting that they may play a role in EGF-induced cell proliferation for this phase of the time course (Fig 4C & 4D). Two other promoters, PHLDA2 and TNFRSF11B show consistently higher expression in the HRG time course than the EGF one (Fig 4A & 4B). The divergence between the two time courses is initially small but grows larger at later time points. The expression profiles of these two promoters suggest that they are important for HRG-induced differentiation for the MCF-7 cells.

**Table 2:** Promoters that are associated with significant fold changes of HRG-induced expression over EGF-induced expression.

Promoter	Gene Symbol	Adjusted P-value Measuring Significance of Change in Fold Change (HRG/EGF)	Regression Coefficient from Linear Model
*chr17*:*39675131*..*39675148*,*-*	KRT15	0.0000	0.06
*chr16*:*28835766*..*28835827*,*+*	N/A	0.0002	0.09
*chr8*:*119964115*..*119964132*,*-*	TNFRSF11B	0.0002	0.18
*chr2*:*120980665*..*120980750*,*-*	TMEM185B	0.0008	0.04
*chr11*:*2950642*..*2950658*,*-*	PHLDA2	0.0014	0.1
*chr10*:*112257656*..*112257671*,*+*	DUSP5	0.0015	-0.02
*chr5*:*137801160*..*137801176*,*+*	EGR1 (TF)	0.0053	-0.21

(TF) after gene symbol identifies the transcription factors.

### Genes mapping uniquely to the generic and stimulus-specific promoters are enriched for different biological processes that control migration, proliferation, apoptosis and cell cycle

Understanding the specific pathways that are used by cells during cell fate decisions can also help shed light on how a transition is regulated. We used the genes that mapped uniquely to promoters in the generic and stimulus-specific groups to identify biological pathways or processes that were enriched exclusively in either group. The motivation was that if a particular pathway was over-represented in the genes controlled in the stimulus-specific group and not the generic group, then this may suggest an important role of this pathway for regulating distinct cell fates or a response to a specific stimuli. We used pathway definitions specified by the Kyoto Encyclopedia of Genes and Genomes (KEGG) [37, 38] and the Gene Ontology Biological Processes (GO:BP) [39]. Fisher’s exact test was used to assess the significance of over-representation of either generic or stimulus-specific promoters in a pathway or biological process. Multiple hypothesis testing was corrected for using the Benjamini-Hochberg method [15] (see Materials and Methods).

The stimulus-specific promoters control genes that were enriched for KEGG pathways involved in focal adhesion, p53 signaling, and MAPK signaling pathways (see S2 Table). Related themes are evident in the significant GO:BP terms where processes related to migration (“positive regulation of cell migration”), cell cycle (“cell cycle”, “cell cycle arrest”), transcription (“positive regulation of transcription from RNA polymerase II promoter”), and cell death (“intrinsic apoptotic signaling pathway in response to DNA damage”) were observed. The genes controlled by generic promoters were also enriched for processes that are fundamental to proper functioning of the cell, and common themes were apparent for cell death (“positive regulation apoptotic processes”, “regulation of apoptotic process”), transcription (“transcription, DNA-dependent”) and the cell cycle (“regulation of cell cycle”) (see S3 Table). It is interesting to note that with respect to proliferation, for the stimulus-specific promoters we saw enrichment of the term “negative regulation of cell proliferation” whereas for the generic promoters, “positive regulation of cell proliferation” was significant (see S2A and S2B Fig). This result implies that generic and stimulus-specific promoters may play opposite roles in regulating cell proliferation. The degree of cell proliferation may be proportional to the ratio of generic versus stimulus-specific gene elements that are being activated and this may in turn also affect cell differentiation.

### Alternative promoters are found in key genes involved in the cell fate transition and had coordinated profiles across the time course

Alternative promoters represent an inbuilt feature of the genome for generating diversity under different cellular conditions. They also provide robustness to ensure that the proper initiation of transcription of critical genes can occur. For the MCF-7 time course, we applied the definition of an alternative promoter as the instance where more than one promoter mapped to the same gene. We found that the expression of 162 genes were controlled by an alternative promoter. Of these 162 genes, 18 were controlled by more than one generic promoter, and 107 were controlled by more than one stimulus-specific promoter. A subset of 37 genes were controlled by a combination of promoters that had expression profiles that were classified into both generic and stimulus-specific groups.

Examples of transcription factors whose expression were controlled by alternative promoters were EGR1 and FOS which were each controlled by seven promoters (see Table 3). For FOS, two of the seven behaved as generic promoters and the remaining five were stimulus-specific. For EGR1, there were four generic promoters and three stimulus-specific promoters mapping to this gene. It is plausible that these transcription factors have dual roles to control the expression of genes needed for both proliferation or differentiation of MCF-7 cells at immediate early times and selectively lead to the transcription of genes required for differentiation following a more sustained expression induced by HRG.

**Table 3:** Genes that are mapped by alternative promoters classified into different generic or stimulus-specific groups.

Gene Symbol	Number of Generic Promoters	Generic Promoters	Number of Stimulus-specific Promoters	Stimulus-specific Proms.
EGR1 (TF)	4	*chr5*:*137804130*..*137804156*,*+//chr5*:*137804357*..*137804390*,*+//chr5*:*137804405*..*137804444*,*+//chr5*:*137804484*..*137804498*,*+*	3	*chr5*:*137800878*..*137800898*,*+//chr5*:*137800912*..*137800941*,*+//chr5*:*137801160*..*137801176*,*+*
FOS (TF)	2	*chr14*:*75746579*..*75746605*,*+//chr14*:*75746705*..*75746720*,*+*	5	*chr14*:*75745523*..*75745537*,*+//chr14*:*75746722*..*75746777*,*+//chr14*:*75746781*..*75746799*,*+//chr14*:*75747250*..*75747267*,*+//chr14*:*75747296*..*75747329*,*+*
ELOVL1	2	*chr1*:*43832006*..*43832027*,*-//chr1*:*43833263*..*43833293*,*-*	1	*chr1*:*43833628*..*43833703*,*-*
PLEC	2	*chr8*:*145027973*..*145027992*,*-//chr8*:*145047688*..*145047704*,*-*	1	*chr8*:*145013711*..*145013786*,*-*
HIST1H2BC	1	*chr6*:*26199709*..*26199720*,*+*	3	*chr6*:*26124147*..*26124168*,*-//chr6*:*26199737*..*26199754*,*+//chr6*:*26273152*..*26273175*,*+*
HIST1H2BE	1	*chr6*:*26199709*..*26199720*,*+*	3	*chr6*:*26124147*..*26124168*,*-//chr6*:*26199737*..*26199754*,*+//chr6*:*26273152*..*26273175*,*+*
HIST1H2BF	1	*chr6*:*26199709*..*26199720*,*+*	3	*chr6*:*26124147*..*26124168*,*-//chr6*:*26199737*..*26199754*,*+//chr6*:*26273152*..*26273175*,*+*
HIST1H2BG	1	*chr6*:*26199709*..*26199720*,*+*	3	*chr6*:*26124147*..*26124168*,*-//chr6*:*26199737*..*26199754*,*+//chr6*:*26273152*..*26273175*,*+*
HIST1H2BI	1	*chr6*:*26199709*..*26199720*,*+*	3	*chr6*:*26124147*..*26124168*,*-//chr6*:*26199737*..*26199754*,*+//chr6*:*26273152*..*26273175*,*+*
SMAD3 (TF)	1	*chr15*:*67418119*..*67418162*,*+*	3	*chr15*:*67358163*..*67358192*,*+//chr15*:*67418047*..*67418093*,*+//chr15*:*67418177*..*67418204*,*+*
HIST1H3A	1	*chr6*:*26197500*..*26197521*,*-*	2	*chr6*:*26020672*..*26020689*,*+//chr6*:*27840112*..*27840133*,*-*
HIST1H3B	1	*chr6*:*26197500*..*26197521*,*-*	2	*chr6*:*26020672*..*26020689*,*+//chr6*:*27840112*..*27840133*,*-*
HIST1H3C	1	*chr6*:*26197500*..*26197521*,*-*	2	*chr6*:*26020672*..*26020689*,*+//chr6*:*27840112*..*27840133*,*-*
HIST1H3D	1	*chr6*:*26197500*..*26197521*,*-*	2	*chr6*:*26020672*..*26020689*,*+//chr6*:*27840112*..*27840133*,*-*
HIST1H3E	1	*chr6*:*26197500*..*26197521*,*-*	2	*chr6*:*26020672*..*26020689*,*+//chr6*:*27840112*..*27840133*,*-*
HIST1H3F	1	*chr6*:*26197500*..*26197521*,*-*	2	*chr6*:*26020672*..*26020689*,*+//chr6*:*27840112*..*27840133*,*-*
HIST1H3G	1	*chr6*:*26197500*..*26197521*,*-*	2	*chr6*:*26020672*..*26020689*,*+//chr6*:*27840112*..*27840133*,*-*
HIST1H3H	1	*chr6*:*26197500*..*26197521*,*-*	2	*chr6*:*26020672*..*26020689*,*+//chr6*:*27840112*..*27840133*,*-*
HIST1H3I	1	*chr6*:*26197500*..*26197521*,*-*	2	*chr6*:*26020672*..*26020689*,*+//chr6*:*27840112*..*27840133*,*-*
HIST1H3J	1	*chr6*:*26197500*..*26197521*,*-*	2	*chr6*:*26020672*..*26020689*,*+//chr6*:*27840112*..*27840133*,*-*
METTL7B	1	*chr12*:*56075495*..*56075509*,*+*	2	*chr12*:*56075432*..*56075444*,*+//chr12*:*56075512*..*56075532*,*+*
ATP1B1	1	*chr1*:*169075919*..*169075940*,*+*	1	*chr1*:*169075554*..*169075571*,*+*
BBC3	1	*chr19*:*47734425*..*47734445*,*-*	1	*chr19*:*47734448*..*47734466*,*-*
BRIP1 (TF)	1	*chr17*:*59940830*..*59940897*,*-*	1	*chr17*:*59940813*..*59940828*,*-*
DDIT4	1	*chr10*:*74034090*..*74034110*,*+*	1	*chr10*:*74033672*..*74033688*,*+*
FAM207A	1	*chr21*:*46359889*..*46359902*,*+*	1	*chr21*:*46359907*..*46359962*,*+*
FAM83H	1	*chr8*:*144815895*..*144815912*,*-*	1	*chr8*:*144815914*..*144815961*,*-*
FOXA1 (TF)	1	*chr14*:*38065203*..*38065215*,*-*	1	*chr14*:*38064495*..*38064506*,*-*
GRB7	1	*chr17*:*37894179*..*37894202*,*+*	1	*chr17*:*37894570*..*37894614*,*+*
GREB1	1	*chr2*:*11679938*..*11679951*,*+*	1	*chr2*:*11679963*..*11679986*,*+*
IFIT5	1	*chr10*:*91174314*..*91174403*,*+*	1	*chr10*:*91174486*..*91174528*,*+*
IRF2BPL	1	*chr14*:*77493956*..*77493999*,*-*	1	*chr14*:*77494141*..*77494170*,*-*
KLF6	1	*chr10*:*3827371*..*3827386*,*-*	1	*chr10*:*3827389*..*3827408*,*-*
MBTPS1	1	*chr16*:*84150492*..*84150558*,*-*	1	*chr16*:*84150410*..*84150456*,*-*
PRR15L	1	*chr17*:*46035113*..*46035124*,*-*	1	*chr17*:*46035187*..*46035260*,*-*
S100A16	1	*chr1*:*153585456*..*153585532*,*-*	1	*chr1*:*153585571*..*153585629*,*-*
WARS	1	*chr14*:*100841675*..*100841691*,*-*	1	*chr14*:*100841940*..*100841958*,*-*

(TF) after a gene symbol identifies transcription factors.

We investigated whether usage of alternative promoters was likely to be for specialization through stage-specific activation or a more general strategy for robustness by having multiple promoters available that can substitute for each other. We reasoned that if two alternative promoters could substitute for each other to activate the same gene, then their expression profiles would be highly correlated during the time course. Alternatively, low or anti-correlated expression profiles indicate that alternative promoters may have roles that differ depending on their activation time. 72.7% of 22 genes that are controlled by more than onegeneric promoter are highly correlated (they have on average, a correlation coefficient > 0.5), and 92.9% of 126 genes are controlled by at least one stimulus-specific promoter. These results suggest that for both generic and stimulus-specific promoters, alternative promoters may substitute for each other and that the multiplicity of promoters mapping to the same gene reflect an inbuilt redundancy and robustness of the cellular system.

### Protein-protein interaction networks show hub activity of key genes in generic and stimulus-specific promoters

**Fig 5:**
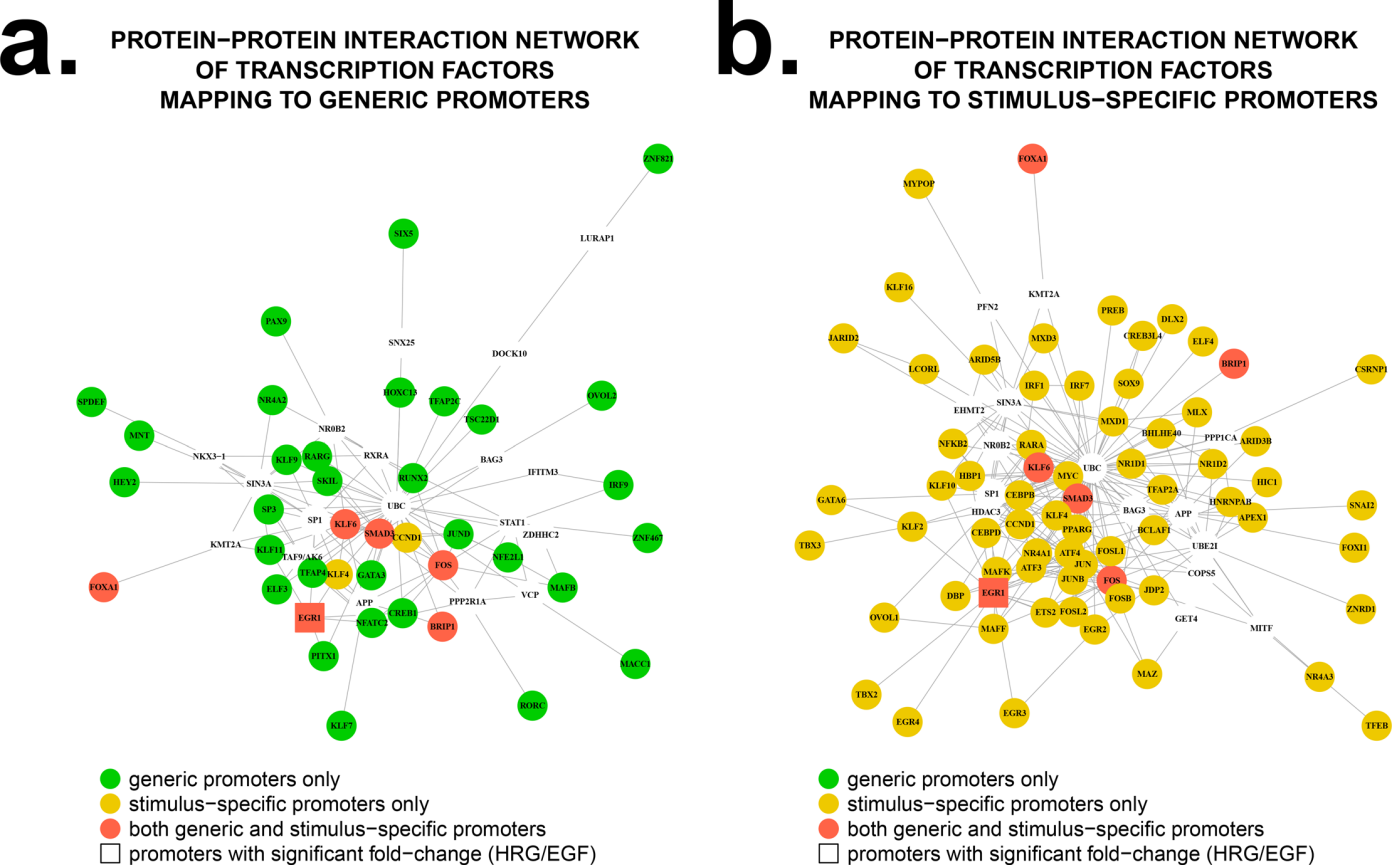
Protein-protein interaction networks (PPI) of genes mapped by the generic and stimulus-specific promoters. **A.** PPI sub-network of shortest paths connecting the 38 transcription factors controlled by generic promoters (32 controlled by generic promoters only, and 6 controlled by alternative promoters classified as either generic or stimulus-specific; note that ZBTB42 (Zinc Finger And BTB Domain Containing 42) does not appear, as it is not listed in iRefIndex). **B.** PPI sub-network connecting the 68 transcription factors controlled by stimulus-specific promoters (62 controlled by stimulus-specific promoters only, and 6 controlled by alternative promoters classified as either generic or stimulus-specific; note that FOXQ1 (forkhead box Q1) does not appear, as it is not listed in iRefIndex). Larger nodes denote TFs, smaller nodes represent non-TF interactors. Green and yellow nodes represent genes mapped by generic promoters and stimulus-specific promoters respectively. Red nodes denote genes that map to both generic and stimulus-specific promoters. Square nodes, like EGF1, identify genes that map to at least one promoter showing significant increments in expression in the HRG over EGF time course.

The transcription of protein-coding genes provides a template for the production of proteins that are the molecules responsible for carrying out fundamental functions in the cell. We recognize that these genes and their corresponding proteins do not operate in isolation but instead interact via defined combinations, and one way in which these that can be represented is through a protein-protein interaction (PPI) network. We investigated whether PPI networks for genes mapping to generic promoters displayed any common or distinct features compared to the PPIs for the stimulus-specific promoters. Because overlaying gene expression data with human protein data is always somewhat of an extrapolation, we restricted our network construction to only the transcription factors that were represented in the generic and stimulus-specific promoter groups (consisting of 38 and 68 transcription factors, respectively). One of the most striking results observed is that the generic and stimulus-specific PPI networks are connected through six common transcription factors that are controlled by alternative promoters, EGR1, FOS, SMAD3 (SMAD family member 3), BRIP1 (BRCA1 unteracting protein C-terminal helicase 1), FOXA1 (forkhead box A1), and KLF6 (Kruppel-Like Factor 6) (Fig 5A and 5B). This resultsuggests these transcriptions factors may play an important role in the MCF-7 time course since alternative promoters exist to control each of their expression. We also see the diversification associated with their usage in the time course, where each transcription factor has both generic and stimulus-specific promoters, suggesting that these transcription factors bridge over the generic (early response) and stimulus-specific promoter (late response) states in the time course. In fact, transcriptional control of FOS is highly complex [40]. For example, the transcriptional activity of SRF (serum response factor), which activates FOS transcription, is known to be regulated in two ways; one is mediated by TCF family cofactor in ERK signal-dependent and another is mediated by the MRTF (myocardian-related transcription factors) family cofactors in a Rho GTPase signal-dependent manner [41]. Alternative FOS promoters presented heremay reflect the different ways in which regulation by SRF can occur (see S3 Fig).

Heatmaps were constructed to highlight the expression profiles of the transcription factors represented in these networks. We see that HRG drives more dramatic expression changes (see S4A and S4B Fig) in both generic and stimulus-specific promoters. The generic transcription factors are mainly expressed at immediate early times, and most stimulus-specific ones are expressed at mid-delayed times. These observations reflected the trends that we had seen more generally for all generic and stimulus-specific promoters. It is possible that following the more stable HRG signals, higher levels of expression of key stimulus-specific transcription factors at mid-delayed times may bring about an active and strong AP-1 complex formation that is responsible for triggering waves of expression that lead to MCF-7 cell differentiation.

### Co-expression networks of transcription factors show more coordinated expression occurs in the HRG-stimulated MCF-7 cells

Regulatory information about the cell fate transition can also be obtained by probing co-expression networks since genes that share similar expression profiles are thought to be regulated by common upstream processes. We constructed co-expression networks for the transcription factors mapping to promoters in the generic and stimulus-specific groups (see Materials and Methods). Networks were constructed under HRG and EGF stimulation separately by joining two genes in the network that had highly correlated expression profiles (S5A–S5D Fig). HRG was associated with a higher degree of co-expression across the time course than EGF and this result was observed for both generic and stimulus-specific promoters. We saw that this effect is more pronounced for the stimulus-specific promoters where there is stronger correlation (and anti-correlation) under HRG than EGF (S6A and S6B Fig). Moreover, for the stimulus-specific promoters that map to transcription factors, HRG is associated with more positive correlation compared to EGF. One possible interpretation of this result is that there is greater coherence and coordination occurring between these transcription factors during cellular differentiation.

### Validation of the main results based on the CAGE data set with two gene expression data sets

To investigate the robustness of the results obtained from the CAGE data set, we applied our analyses to two other gene expression data sets from a microarray experiment [10] and a qPCR experiment [6]. Both data sets were profiled using the MCF-7 cell line that had been exposed to EGF and HRG with data points sampled over a time course that overlapped with the design of the CAGE experiment. Overall, we identified a statistically significant overlap in the lists of stimulus-specific and generic genes between the microarray and CAGE data sets (P-value = 1.38×10^−6^). The overlap with the qPCR data set was not statistically significant (P-value = 0.738), and this may have been due to the fact that only a targeted set of genes (2,352 transcription factors) were profiled in the qPCR experiment whereas the CAGE data set was at genome-wide resolution. The frequency at which time points were sampled was also sparser in the qPCR data set than the CAGE data set. Despite the lack of statistical significance, we observed consistent sets of genes between the qPCR and CAGE data sets that were classified as generic (32 genes) and stimulus-specific (41 genes). Amongst these lists, we detected transcription factors that were key regulators of EGF and HRG signaling such as EGR1, FOS, SP3 (Sp3 transcription factor), SMAD3, and BRIP1 for the generic genes, and FOSL2 (FOS-like antigen 2), FOXA1, and JUN (jun proto-oncogene) for the stimulus-specific genes. We inspected the expression activity for several genes from both stimulus-specific and generic sets in both CAGE and microarrays data sets and found that some profiles were qualitatively consistent. We also compared the functional enrichment results of the stimulus-specific and generic gene lists, and found a subset of significant GO terms and KEGG pathways that were enriched in both microarray and CAGE data sets. Our results from this comparison are described in more detail in S1 Text. Overall, while there are some data-specific differences, we found many consistencies in the main results observed from the CAGE data set with the microarray and qPCR data sets.

Additionally, it is worthwhile highlighting that although different computational methods were used to analyze the qPCR and microarray data sets in the original papers from the trajectory models we have applied to the CAGE data set, similar conclusions were observed in the results we obtained [6, 10]. In both qPCR and microarray data sets, subsets of gene sets were identified that followed patterns that were similar to the stimuli-specific and common, generic definitions that we have adopted in this paper. The observation that different computational methods applied to data sets generated under two different gene expression platforms have identified results that are similar to the major conclusions derived from the CAGE data set lends support to the robustness of our findings.

## Discussion

It is unsurprising that the balance between generic and stimulus-specific promoters was so skewed towards the latter group (70% versus 30%) because not only were the MCF-7 cells exposed to different stimuli but as a result, they also transition to different cell fates. As a result of the MCF-7 example that we have chosen, the stimulus-specific trajectory model captures the promoters that have both cell fate-specific and stimulus-specific expression responses of the cellular system. The generic trajectory model instead captures the promoters with behavior that is coordinated between both stimuli. We would therefore expect that a larger number of promoters are needed to participate in the transition of cells towards different fates as well as recruitment to respond to different stimuli.

Our analysis indicated that both generic and stimulus-specific promoters target AP-1 complex including FOS. FOS contains a conserved DEF domain, susceptible to phosphorylation by ERK, and this domain makes FOS a good sensor for an upstream ERK signal [42, 43]. An HRG-induced sustained ERK signal mediates phosphorylation of FOS protein, under a more potent and stable control than that observed under EGF treatment [7]. Differences in the expression levels of IEGs like FOS have a big impact on successive transcriptional events, as they are often hubs in regulatory networks. These immediate early quantitative differences at the transcription factor level will, in turn, be converted into qualitatively different induction in successive transcriptional events. This would inhibit the original upstream signal by negative feedback once it has become unnecessary as seen in the case of FHL2.

As for EGR1, we found this gene was represented by both generic and stimulus-specific promoters. An earlier study showed using two EGF pulse-stimulated 184A1 human mammary cells that both p53-dependent restraining processes followed by the second pulse elimination of the suppressive action of p53 via the PI3K/AKT pathway as well as ERK-EGR1 threshold mechanism is necessary for S phase entry [44]. In addition, another study using MCF10A mammary cells that involve EGR1 and the ERK-ERF axis to drive mammary cell migration in response to EGF [45]. The study is partially consistent with our current observation that the EGF- and HRG-mediated ERK-AP-1 axis and EGR1 are central components of cell divergence of MCF-7 mammary tumor cells, and that the ERK signal is dynamically regulated by the transcriptional negative feedback through FHL2 or DUSP5, a MAPK phosphatase.

The observation of alternative promoters that map to the same genes demonstrated how it is possible to control the one gene via multiple signals. Amongst the alternative promoters that we identified, we saw that the majority featured alternative promoters that had stimulus-specific expression and suggested that promoters mapping to the same gene were all being expressed in a divergent manner between different stimuli. The observation that another group of genes (37 out of 162) had alternative promoters that were represented in both generic and stimulus-specific groups was interesting as this highlights the divergent nature of how genes are expressed and controlled across their corresponding promoters regions. Other CAGE studies have exposed the level of discordance that can occur for different promoters that map to the same gene, suggesting that to really understand transcription and its functional consequences, we need to move to the resolution of promoters as opposed to genes.

The comparison of PPI networks for generic and stimulus-specific promoters reflect the convergence of overlapping components in the signaling pathways, specifically for the six transcription common TFs (EGR1, FOS, SMAD3, BRIP1, FOXA1, and KLF6). We saw many PPIs that were unique to the generic versus stimulus-specific networks which was consistent with the idea that these genes and their proteins are required in different stages and abundances during the time course, depending on the role they play in the cell fate transition. However, the six common transcription factors mark components of the signaling pathways where their usage, at the expression level at least, occurs in both a generic and stimulus-specific manner at different timing regulated by alternative promoters. The result suggests that regulatory mechanisms of these transcription factors are in fact very diverse and precisely controlled in a time-dependent manner, and that divergent regulatory events are integrated as a core network that functions to act as a robust axis to drive cell determination.

## Supporting Information

S1 Fig Heatmaps showing patterns of expression for the highly-expressed generic and stimulus-specific promoters. These values span the whole range of generic and stimulus-specific promoters values for **A.** 49 generic promoters and **B.** 100 stimulus-specific promoters, identified as being the most highly-expressed (average expression value in either EGF or HRG treatment was greater than the 85% percentile of values under the corresponding treatment). There are many more genes controlled by stimulus-specific promoters highly expressed, particularly under the HRG treatment and at mid-delayed times.
(EPS)

S2 Fig Heatmaps showing the expression profiles of promoters that map to genes involved in controlling cell proliferation. **A.** There were 16 generic promoters that mapped to genes in the GO term ‘negative regulation of cell proliferation’. **B.** There were 21 stimulus-specific promoters that mapped to genes in the GO term ‘positive regulation of cell proliferation’.
(EPS)

S3 Fig Heatmap showing the expression profiles of seven alternative promoters that all map to FOS. (EPS)

S4 Fig Heatmaps showing the expression profiles of the A. generic or B. stimulus-specific promoters that map to transcription factors (scaled by promoter). As observed for the whole set of generic and stimulus-specific promoters, HRG drives more dramatic expression changes; while generic transcription factors are mainly expressed at immediate early times, most stimulus-specific ones are expressed at mid-delayed times.
(EPS)

S5 Fig Co-expression networks in EGF and HRG time courses A. and C. for the 38 transcription factors controlled by generic promoters and B. and D. the 68 transcription factors controlled by stimulus-specific promoters. Each node represents a gene, and the edges join genes that are highly-correlated during the time course (|correlation coefficient| > 0.95). The color of an edge indicates the strength of correlation, ranging from blue (negative correlation) to white (uncorrelated) to red (positively correlated). We see more dramatic co-expression in the HRG time course compared to the EGF time course, and this is observed more readily for the subset of stimulus-specific transcription factors.
(EPS)

S6 Fig Density distributions of the correlation coefficients for the transcription factor pairs for the A. 38 transcription factors controlled by generic promoters and the B. 68 transcription factors controlled by stimulus-specific promoters under EGF (red) and HRG (blue) treatments. While the expression of the majority of transcription factor pairs is uncorrelated under EGF treatment (describing an almost normal distribution), the expression of the majority of transcription factor pairs is highly correlated, either positively or negatively (describing a bimodal distribution), under HRG. These differences in correlated expression for both generic and stimulus-specific transcription factors are particularly striking for stimulus-specific transcription factors, which tend to be more positively correlated.
(EPS)

S1 Table List of FANTOM Consortium members. (DOCX)

S2 Table Statistically significant GO and KEGG terms for the generic genes identified from the CAGE data set. Statistical significance was defined as adjusted P-value < 0.05 using the Benjamini-Hochberg method. No KEGG pathway was found to meet statistical significant.
(DOCX)

S3 Table Statistically significant GO and KEGG terms for the stimulus-specific genes identified from the CAGE data set. Statistical significance was defined as adjusted P-value < 0.05 using the Benjamini-Hochberg method.
(DOCX)

S1 Text Validation of CAGE results with other gene expression data sets. (DOCX)
