# Supplementary material for: Application of Gene Expression Trajectories Initiated from ErbB Receptor Activation Highlights the Dynamics of Divergent Promoter Usage
Source: PLoS One. 2015 Dec 14;10(12):e0144176. doi: 10.1371/journal.pone.0144176 (PMC4682858; doi:10.1371/journal.pone.0144176)
Supplement: S2 Table — Statistical significance was defined as adjusted P-value < 0.05 using the Benjamini-Hochberg method. No KEGG pathway was found to meet statistical significant. (DOCX) [file pone.0144176.s008.docx]

**Table S2. Statistically significant GO and KEGG terms for the generic genes identified from the CAGE data set.** Statistical significance was defined as adjusted P-value < 0.05 using the Benjamini-Hochberg method. No KEGG pathway was found to meet statistical significance.

| **Genes group** | **Term group** | **ID** | **Term** | **Genes in univ. w/ term** | **Input genes w/ term** | **Adj. P-value** | **Genes in universe** | **Input genes** |
| --- | --- | --- | --- | --- | --- | --- | --- | --- |
| ALL Generic genes | GO:BP | GO:0043065 | *positive regulation of apoptotic process* | 283 | 15 | 6.41E-003 | 18585 | 256 |
|  |  | GO:0045682 | *regulation of epidermis development* | 4 | 3 | 6.41E-003 | 18585 | 256 |
|  |  | GO:0000122 | *negative regulation of transcription from RNA polymerase II promoter* | 656 | 24 | 6.41E-003 | 18585 | 256 |
|  |  | GO:0045893 | *positive regulation of transcription, DNA-templated* | 495 | 20 | 6.41E-003 | 18585 | 256 |
|  |  | GO:0043966 | *histone H3 acetylation* | 44 | 6 | 8.09E-003 | 18585 | 256 |
|  |  | GO:0008285 | *negative regulation of cell proliferation* | 381 | 16 | 1.89E-002 | 18585 | 256 |
|  |  | GO:0006366 | *transcription from RNA polymerase II promoter* | 487 | 18 | 2.82E-002 | 18585 | 256 |
|  |  | GO:0010719 | *negative regulation of epithelial to mesenchymal transition* | 21 | 4 | 2.82E-002 | 18585 | 256 |
|  |  | GO:0045944 | *positive regulation of transcription from RNA polymerase II promoter* | 873 | 26 | 2.82E-002 | 18585 | 256 |
|  |  | GO:0032060 | *bleb assembly* | 9 | 3 | 2.82E-002 | 18585 | 256 |
|  | GO:CC | GO:0005654 | *nucleoplasm* | 2534 | 69 | 2.72E-006 | 18585 | 256 |
|  |  | GO:0005634 | *nucleus* | 4981 | 104 | 1.16E-004 | 18585 | 256 |
|  |  | GO:0034045 | *pre-autophagosomal structure membrane* | 15 | 4 | 3.46E-003 | 18585 | 256 |
|  |  | GO:0005737 | *cytoplasm* | 4677 | 91 | 7.85E-003 | 18585 | 256 |
|  |  | GO:0097632 | *extrinsic component of pre-autophagosomal structure membrane* | 2 | 2 | 9.22E-003 | 18585 | 256 |
|  |  | GO:0097629 | *extrinsic component of omegasome membrane* | 3 | 2 | 2.28E-002 | 18585 | 256 |
|  |  | GO:0030914 | *STAGA complex* | 13 | 3 | 2.32E-002 | 18585 | 256 |
|  |  | GO:0048471 | *perinuclear region of cytoplasm* | 567 | 18 | 2.80E-002 | 18585 | 256 |
|  | GO:MF | GO:0005515 | *protein binding* | 8358 | 158 | 1.48E-005 | 18585 | 256 |
|  |  | GO:0004402 | *histone acetyltransferase activity* | 42 | 7 | 2.62E-004 | 18585 | 256 |
|  |  | GO:0003713 | *transcription coactivator activity* | 244 | 13 | 4.02E-003 | 18585 | 256 |
|  |  | GO:0001077 | *RNA polymerase II core promoter proximal region sequence-specific DNA binding transcription factor activity involved in positive regulation of transcription* | 188 | 11 | 5.21E-003 | 18585 | 256 |
|  |  | GO:0003700 | *sequence-specific DNA binding transcription factor activity* | 825 | 24 | 3.19E-002 | 18585 | 256 |
| Generic genes + Coeff. | GO:BP | GO:0009615 | *response to virus* | 111 | 6 | 3.38E-002 | 18585 | 124 |
|  |  | GO:0019065 | *receptor-mediated endocytosis of virus by host cell* | 3 | 2 | 3.38E-002 | 18585 | 124 |
|  |  | GO:0045061 | *thymic T cell selection* | 3 | 2 | 3.38E-002 | 18585 | 124 |
|  |  | GO:0015949 | *nucleobase-containing small molecule interconversion* | 18 | 3 | 3.38E-002 | 18585 | 124 |
|  |  | GO:0045064 | *T-helper 2 cell differentiation* | 4 | 2 | 3.38E-002 | 18585 | 124 |
|  |  | GO:0070836 | *caveola assembly* | 4 | 2 | 3.38E-002 | 18585 | 124 |
|  | GO:CC | GO:0070062 | *extracellular vesicular exosome* | 2759 | 34 | 3.18E-002 | 18585 | 124 |
|  |  | GO:0005829 | *cytosol* | 2797 | 33 | 4.53E-002 | 18585 | 124 |
|  | GO:MF | GO:0005515 | *protein binding* | 8358 | 79 | 3.92E-003 | 18585 | 124 |
|  |  | GO:0001077 | *RNA polymerase II core promoter proximal region sequence-specific DNA binding transcription factor activity involved in positive regulation of transcription* | 188 | 8 | 3.92E-003 | 18585 | 124 |
| Generic genes - Coeff. | GO:BP | GO:0043966 | *histone H3 acetylation* | 44 | 5 | 6.05E-003 | 18585 | 131 |
|  |  | GO:0051726 | *regulation of cell cycle* | 112 | 7 | 6.05E-003 | 18585 | 131 |
|  |  | GO:0007623 | *circadian rhythm* | 91 | 6 | 1.25E-002 | 18585 | 131 |
|  |  | GO:0003199 | *endocardial cushion to mesenchymal transition involved in heart valve formation* | 3 | 2 | 2.65E-002 | 18585 | 131 |
|  |  | GO:0006366 | *transcription from RNA polymerase II promoter* | 487 | 12 | 2.65E-002 | 18585 | 131 |
|  |  | GO:0045893 | *positive regulation of transcription, DNA-templated* | 495 | 12 | 2.65E-002 | 18585 | 131 |
|  |  | GO:0000122 | *negative regulation of transcription from RNA polymerase II promoter* | 656 | 14 | 2.65E-002 | 18585 | 131 |
|  |  | GO:0000045 | *autophagic vacuole assembly* | 45 | 4 | 2.95E-002 | 18585 | 131 |
|  |  | GO:0010719 | *negative regulation of epithelial to mesenchymal transition* | 21 | 3 | 3.87E-002 | 18585 | 131 |
|  | GO:CC | GO:0005654 | *nucleoplasm* | 2534 | 39 | 1.95E-004 | 18585 | 131 |
|  |  | GO:0034045 | *pre-autophagosomal structure membrane* | 15 | 4 | 2.55E-004 | 18585 | 131 |
|  |  | GO:0005634 | *nucleus* | 4981 | 58 | 6.59E-004 | 18585 | 131 |
|  |  | GO:0097632 | *extrinsic component of pre-autophagosomal structure membrane* | 2 | 2 | 2.07E-003 | 18585 | 131 |
|  |  | GO:0097629 | *extrinsic component of omegasome membrane* | 3 | 2 | 4.95E-003 | 18585 | 131 |
|  |  | GO:0030915 | *Smc5-Smc6 complex* | 7 | 2 | 2.83E-002 | 18585 | 131 |
|  | GO:MF | GO:0004402 | *histone acetyltransferase activity* | 42 | 6 | 9.87E-005 | 18585 | 131 |
|  |  | GO:0003713 | *transcription coactivator activity* | 244 | 10 | 9.64E-004 | 18585 | 131 |
|  |  | GO:0005086 | *ARF guanyl-nucleotide exchange factor activity* | 18 | 3 | 1.83E-002 | 18585 | 131 |
|  |  | GO:0005515 | *protein binding* | 8358 | 78 | 2.87E-002 | 18585 | 131 |
