# Supplementary material for: Application of Gene Expression Trajectories Initiated from ErbB Receptor Activation Highlights the Dynamics of Divergent Promoter Usage
Source: PLoS One. 2015 Dec 14;10(12):e0144176. doi: 10.1371/journal.pone.0144176 (PMC4682858; doi:10.1371/journal.pone.0144176)
Supplement: S3 Table — Statistical significance was defined as adjusted P-value < 0.05 using the Benjamini-Hochberg method. (DOCX) [file pone.0144176.s009.docx]

**Table S3.** **Statistically significant GO and KEGG terms for the stimulus-specific genes identified from the CAGE data set.** Statistical significance was defined as adjusted P-value < 0.05 using the Benjamini-Hochberg method.

| **Genes group** | **Term group** | **ID** | **Term** | **Genes in univ. w/ term** | **Input genes w/ term** | **Adj. P-value** | **Genes in universe** | **Input genes** |
| --- | --- | --- | --- | --- | --- | --- | --- | --- |
| ALL Stimulus-specific genes | KEGG | 5322 | *Systemic lupus erythematosus* | 136 | 21 | 2.71E-004 | 5869 | 284 |
|  |  | 4510 | *Focal adhesion* | 200 | 26 | 2.71E-004 | 5869 | 284 |
|  |  | 4115 | *p53 signaling pathway* | 68 | 13 | 1.01E-003 | 5869 | 284 |
|  |  | 5130 | *Pathogenic Escherichia coli infection* | 56 | 11 | 2.62E-003 | 5869 | 284 |
|  |  | 4010 | *MAPK signaling pathway* | 268 | 28 | 2.88E-003 | 5869 | 284 |
|  |  | 5219 | *Bladder cancer* | 42 | 8 | 2.24E-002 | 5869 | 284 |
|  |  | 4380 | *Osteoclast differentiation* | 128 | 15 | 2.89E-002 | 5869 | 284 |
|  |  | 4670 | *Leukocyte transendothelial migration* | 116 | 14 | 2.89E-002 | 5869 | 284 |
|  |  | 5131 | *Shigellosis* | 61 | 9 | 4.73E-002 | 5869 | 284 |
|  | GO:BP | GO:0045653 | *negative regulation of megakaryocyte differentiation* | 16 | 14 | 1.24E-014 | 13178 | 583 |
|  |  | GO:0006336 | *DNA replication-independent nucleosome assembly* | 26 | 14 | 3.31E-010 | 13178 | 583 |
|  |  | GO:0051290 | *protein heterotetramerization* | 28 | 14 | 8.44E-010 | 13178 | 583 |
|  |  | GO:0006335 | *DNA replication-dependent nucleosome assembly* | 32 | 14 | 6.31E-009 | 13178 | 583 |
|  |  | GO:0006334 | *nucleosome assembly* | 46 | 15 | 1.38E-007 | 13178 | 583 |
|  |  | GO:0030335 | *positive regulation of cell migration* | 90 | 19 | 2.09E-006 | 13178 | 583 |
|  |  | GO:0045944 | *positive regulation of transcription from RNA polymerase II promoter* | 483 | 43 | 1.65E-003 | 13178 | 583 |
|  |  | GO:2000210 | *positive regulation of anoikis* | 5 | 4 | 2.65E-003 | 13178 | 583 |
|  |  | GO:0000122 | *negative regulation of transcription from RNA polymerase II promoter* | 296 | 30 | 2.65E-003 | 13178 | 583 |
|  |  | GO:0007179 | *transforming growth factor beta receptor signaling pathway* | 32 | 8 | 6.09E-003 | 13178 | 583 |
|  |  | GO:0007050 | *cell cycle arrest* | 41 | 9 | 6.09E-003 | 13178 | 583 |
|  |  | GO:0042149 | *cellular response to glucose starvation* | 11 | 5 | 6.09E-003 | 13178 | 583 |
|  |  | GO:0030801 | *positive regulation of cyclic nucleotide metabolic process* | 3 | 3 | 7.86E-003 | 13178 | 583 |
|  |  | GO:0008284 | *positive regulation of cell proliferation* | 191 | 21 | 9.40E-003 | 13178 | 583 |
|  |  | GO:0045892 | *negative regulation of transcription, DNA-templated* | 296 | 28 | 1.00E-002 | 13178 | 583 |
|  |  | GO:0070527 | *platelet aggregation* | 28 | 7 | 1.25E-002 | 13178 | 583 |
|  |  | GO:0043066 | *negative regulation of apoptotic process* | 198 | 21 | 1.29E-002 | 13178 | 583 |
|  |  | GO:0007229 | *integrin-mediated signaling pathway* | 29 | 7 | 1.40E-002 | 13178 | 583 |
|  |  | GO:0008630 | *intrinsic apoptotic signaling pathway in response to DNA damage* | 14 | 5 | 1.49E-002 | 13178 | 583 |
|  |  | GO:0006468 | *protein phosphorylation* | 205 | 21 | 1.77E-002 | 13178 | 583 |
|  |  | GO:0007184 | *SMAD protein import into nucleus* | 4 | 3 | 1.80E-002 | 13178 | 583 |
|  |  | GO:0051343 | *positive regulation of cyclic-nucleotide phosphodiesterase activity* | 4 | 3 | 1.80E-002 | 13178 | 583 |
|  |  | GO:0035307 | *positive regulation of protein dephosphorylation* | 16 | 5 | 2.39E-002 | 13178 | 583 |
|  |  | GO:2000379 | *positive regulation of reactive oxygen species metabolic process* | 16 | 5 | 2.39E-002 | 13178 | 583 |
|  |  | GO:0032516 | *positive regulation of phosphoprotein phosphatase activity* | 10 | 4 | 3.05E-002 | 13178 | 583 |
|  |  | GO:0035767 | *endothelial cell chemotaxis* | 5 | 3 | 3.54E-002 | 13178 | 583 |
|  |  | GO:0045742 | *positive regulation of epidermal growth factor receptor signaling pathway* | 5 | 3 | 3.54E-002 | 13178 | 583 |
|  | GO:CC | GO:0005634 | *nucleus* | 2566 | 186 | 4.77E-011 | 13178 | 583 |
|  |  | GO:0000786 | *nucleosome* | 33 | 14 | 3.03E-009 | 13178 | 583 |
|  |  | GO:0000228 | *nuclear chromosome* | 39 | 15 | 3.03E-009 | 13178 | 583 |
|  |  | GO:0005925 | *focal adhesion* | 339 | 44 | 7.08E-009 | 13178 | 583 |
|  |  | GO:0001725 | *stress fiber* | 29 | 9 | 1.31E-004 | 13178 | 583 |
|  |  | GO:0005730 | *nucleolus* | 606 | 50 | 5.97E-004 | 13178 | 583 |
|  |  | GO:0043234 | *protein complex* | 224 | 25 | 6.68E-004 | 13178 | 583 |
|  |  | GO:0000407 | *pre-autophagosomal structure* | 5 | 3 | 2.40E-002 | 13178 | 583 |
|  | GO:MF | GO:0044822 | *poly(A) RNA binding* | 1109 | 95 | 7.36E-008 | 13178 | 583 |
|  |  | GO:0005515 | *protein binding* | 8358 | 426 | 5.78E-005 | 13178 | 583 |
|  |  | GO:0042393 | *histone binding* | 83 | 16 | 7.99E-005 | 13178 | 583 |
|  |  | GO:0031997 | *N-terminal myristoylation domain binding* | 3 | 3 | 8.98E-003 | 13178 | 583 |
|  |  | GO:0034714 | *type III transforming growth factor beta receptor binding* | 4 | 3 | 2.06E-002 | 13178 | 583 |
|  |  | GO:0072542 | *protein phosphatase activator activity* | 4 | 3 | 2.06E-002 | 13178 | 583 |
|  |  | GO:0001046 | *core promoter sequence-specific DNA binding* | 15 | 5 | 2.06E-002 | 13178 | 583 |
| Stimulus-specific genes + Coeff. | KEGG | 5322 | *Systemic lupus erythematosus* | 136 | 21 | 2.24E-006 | 5869 | 213 |
|  |  | 4510 | *Focal adhesion* | 200 | 23 | 5.96E-005 | 5869 | 213 |
|  |  | 5130 | *Pathogenic Escherichia coli infection* | 56 | 11 | 2.23E-004 | 5869 | 213 |
|  |  | 5214 | *Glioma* | 65 | 9 | 2.14E-002 | 5869 | 213 |
|  |  | 4115 | *p53 signaling pathway* | 68 | 9 | 2.40E-002 | 5869 | 213 |
|  |  | 4670 | *Leukocyte transendothelial migration* | 116 | 12 | 2.67E-002 | 5869 | 213 |
|  |  | 5131 | *Shigellosis* | 61 | 8 | 3.60E-002 | 5869 | 213 |
|  | GO:BP | GO:0045653 | *negative regulation of megakaryocyte differentiation* | 16 | 14 | 4.50E-017 | 13178 | 400 |
|  |  | GO:0006336 | *DNA replication-independent nucleosome assembly* | 26 | 14 | 1.37E-012 | 13178 | 400 |
|  |  | GO:0051290 | *protein heterotetramerization* | 28 | 14 | 3.59E-012 | 13178 | 400 |
|  |  | GO:0006335 | *DNA replication-dependent nucleosome assembly* | 32 | 14 | 2.83E-011 | 13178 | 400 |
|  |  | GO:0006334 | *nucleosome assembly* | 46 | 15 | 5.03E-010 | 13178 | 400 |
|  |  | GO:0030335 | *positive regulation of cell migration* | 90 | 15 | 1.11E-005 | 13178 | 400 |
|  |  | GO:0007050 | *cell cycle arrest* | 41 | 8 | 3.05E-003 | 13178 | 400 |
|  |  | GO:0030801 | *positive regulation of cyclic nucleotide metabolic process* | 3 | 3 | 3.05E-003 | 13178 | 400 |
|  |  | GO:0051343 | *positive regulation of cyclic-nucleotide phosphodiesterase activity* | 4 | 3 | 9.95E-003 | 13178 | 400 |
|  |  | GO:0043066 | *negative regulation of apoptotic process* | 198 | 17 | 9.95E-003 | 13178 | 400 |
|  |  | GO:0070527 | *platelet aggregation* | 28 | 6 | 1.29E-002 | 13178 | 400 |
|  |  | GO:0007229 | *integrin-mediated signaling pathway* | 29 | 6 | 1.40E-002 | 13178 | 400 |
|  |  | GO:0051897 | *positive regulation of protein kinase B signaling* | 41 | 7 | 1.40E-002 | 13178 | 400 |
|  |  | GO:0042149 | *cellular response to glucose starvation* | 11 | 4 | 1.46E-002 | 13178 | 400 |
|  |  | GO:2000210 | *positive regulation of anoikis* | 5 | 3 | 1.55E-002 | 13178 | 400 |
|  |  | GO:0007179 | *transforming growth factor beta receptor signaling pathway* | 32 | 6 | 1.92E-002 | 13178 | 400 |
|  |  | GO:0060316 | *positive regulation of ryanodine-sensitive calcium-release channel activity* | 6 | 3 | 2.63E-002 | 13178 | 400 |
|  |  | GO:0030036 | *actin cytoskeleton organization* | 23 | 5 | 2.63E-002 | 13178 | 400 |
|  |  | GO:0043536 | *positive regulation of blood vessel endothelial cell migration* | 14 | 4 | 3.04E-002 | 13178 | 400 |
|  |  | GO:0032911 | *negative regulation of transforming growth factor beta1 production* | 2 | 2 | 3.70E-002 | 13178 | 400 |
|  |  | GO:0051795 | *positive regulation of catagen* | 2 | 2 | 3.70E-002 | 13178 | 400 |
|  |  | GO:0045766 | *positive regulation of angiogenesis* | 52 | 7 | 3.70E-002 | 13178 | 400 |
|  |  | GO:0035307 | *positive regulation of protein dephosphorylation* | 16 | 4 | 4.17E-002 | 13178 | 400 |
|  |  | GO:2000379 | *positive regulation of reactive oxygen species metabolic process* | 16 | 4 | 4.17E-002 | 13178 | 400 |
|  |  | GO:0001666 | *response to hypoxia* | 41 | 6 | 4.36E-002 | 13178 | 400 |
|  |  | GO:0043154 | *negative regulation of cysteine-type endopeptidase activity involved in apoptotic process* | 41 | 6 | 4.36E-002 | 13178 | 400 |
|  |  | GO:0005513 | *detection of calcium ion* | 8 | 3 | 4.36E-002 | 13178 | 400 |
|  |  | GO:0010880 | *regulation of release of sequestered calcium ion into cytosol by sarcoplasmic reticulum* | 8 | 3 | 4.36E-002 | 13178 | 400 |
|  |  | GO:0030838 | *positive regulation of actin filament polymerization* | 17 | 4 | 4.41E-002 | 13178 | 400 |
|  |  | GO:0001934 | *positive regulation of protein phosphorylation* | 73 | 8 | 4.64E-002 | 13178 | 400 |
|  |  | GO:0010595 | *positive regulation of endothelial cell migration* | 29 | 5 | 4.64E-002 | 13178 | 400 |
|  | GO:CC | GO:0000786 | *nucleosome* | 33 | 14 | 4.05E-011 | 13178 | 400 |
|  |  | GO:0000228 | *nuclear chromosome* | 39 | 14 | 3.16E-010 | 13178 | 400 |
|  |  | GO:0005925 | *focal adhesion* | 339 | 36 | 3.35E-009 | 13178 | 400 |
|  |  | GO:0005634 | *nucleus* | 2566 | 121 | 4.35E-006 | 13178 | 400 |
|  |  | GO:0001725 | *stress fiber* | 29 | 9 | 4.35E-006 | 13178 | 400 |
|  |  | GO:0016020 | *membrane* | 1353 | 68 | 5.91E-004 | 13178 | 400 |
|  |  | GO:0043234 | *protein complex* | 224 | 19 | 1.42E-003 | 13178 | 400 |
|  |  | GO:0070062 | *extracellular vesicular exosome* | 2757 | 114 | 3.73E-003 | 13178 | 400 |
|  |  | GO:0000307 | *cyclin-dependent protein kinase holoenzyme complex* | 7 | 3 | 1.65E-002 | 13178 | 400 |
|  |  | GO:0030017 | *sarcomere* | 7 | 3 | 1.65E-002 | 13178 | 400 |
|  | GO:MF | GO:0044822 | *poly(A) RNA binding* | 1109 | 73 | 4.84E-008 | 13178 | 400 |
|  |  | GO:0042393 | *histone binding* | 83 | 14 | 2.80E-005 | 13178 | 400 |
|  |  | GO:0031997 | *N-terminal myristoylation domain binding* | 3 | 3 | 2.93E-003 | 13178 | 400 |
|  |  | GO:0034714 | *type III transforming growth factor beta receptor binding* | 4 | 3 | 6.88E-003 | 13178 | 400 |
|  |  | GO:0072542 | *protein phosphatase activator activity* | 4 | 3 | 6.88E-003 | 13178 | 400 |
| Stimulus-specific genes - Coeff. | KEGG | 4010 | *MAPK signaling pathway* | 268 | 12 | 4.12E-003 | 5869 | 68 |
|  | GO:BP | GO:0000122 | *negative regulation of transcription from RNA polymerase II promoter* | 296 | 14 | 1.11E-002 | 13178 | 174 |
|  |  | GO:2000144 | *positive regulation of DNA-templated transcription, initiation* | 6 | 3 | 1.11E-002 | 13178 | 174 |
|  |  | GO:0045944 | *positive regulation of transcription from RNA polymerase II promoter* | 483 | 18 | 1.11E-002 | 13178 | 174 |
|  |  | GO:0006468 | *protein phosphorylation* | 205 | 11 | 1.11E-002 | 13178 | 174 |
|  |  | GO:0097192 | *extrinsic apoptotic signaling pathway in absence of ligand* | 3 | 2 | 4.11E-002 | 13178 | 174 |
|  |  | GO:0035556 | *intracellular signal transduction* | 77 | 6 | 4.11E-002 | 13178 | 174 |
|  |  | GO:0045892 | *negative regulation of transcription, DNA-templated* | 296 | 12 | 4.11E-002 | 13178 | 174 |
|  | GO:CC | GO:0005634 | *nucleus* | 2566 | 63 | 2.02E-005 | 13178 | 174 |
|  |  | GO:0005654 | *nucleoplasm* | 1806 | 44 | 1.91E-003 | 13178 | 174 |
|  |  | GO:0005730 | *nucleolus* | 606 | 19 | 1.76E-002 | 13178 | 174 |
|  | GO:MF | GO:0005515 | *protein binding* | 8358 | 135 | 6.79E-003 | 13178 | 174 |
|  |  | GO:0003713 | *transcription coactivator activity* | 106 | 8 | 7.07E-003 | 13178 | 174 |
|  |  | GO:0004674 | *protein serine/threonine kinase activity* | 163 | 9 | 1.86E-002 | 13178 | 174 |
