# Supplementary material for: Application of Gene Expression Trajectories Initiated from ErbB Receptor Activation Highlights the Dynamics of Divergent Promoter Usage
Source: PLoS One. 2015 Dec 14;10(12):e0144176. doi: 10.1371/journal.pone.0144176 (PMC4682858; doi:10.1371/journal.pone.0144176)
Supplement: S1 Text — (DOCX) [file pone.0144176.s010.docx]

**S1 Text: Validation of CAGE results with other gene expression data sets**

To investigate whether the results identified from the CAGE data were observed in other gene expression data sets, we applied the same set of analyses to a qRT-PCR data set and microarray data set that were both generated by the Okada-Hatakeyama lab. These data sets were profiled on the same MCF-7 cell line and exposed to HRG and EGF over time courses that overlapped with the design of the CAGE time course (Table 1).

Both data sets were described in

- Saeki Y, Endo T, Ide K, Nagashima T et al. Ligand-specific sequential regulation of transcription factors for differentiation of MCF-7 cells. BMC Genomics 2009 Nov 20;10:545. PMID: 19925682

**Table 1.** Description of the data sets used for validation of the CAGE results.

|  | **Number of Genes Profiled** | **Time Points** | **Design** | **Total Number of Samples** |
| --- | --- | --- | --- | --- |
| Microarray Data Set | 22, 277 | 10min, 15min, 20min, 30min, 45min, 1hr, 1.5hr, 2hr, 3hr, 4hr, 6hr, 8hr, 12hr, 24hr, 36hr, 48hr, 72hr after exposure to HRG or EGF (17 times points in total) | Control Sample + 17 time points (two replicates per time point). | 69 samples |
| qPCR Data Set | 2, 352 | 0hr, 0.5hr, 1hr, 2hr, 4hr and 6 hr after exposure to HRG or EGF (6 time points in total) | 6 time points (one sample per time point) | 12 samples |

*Comparing sets of stimulus-specific and generic genes with those identified by the CAGE data set.*

We first identified the genes that had stimulus-specific expression profiles (highly divergent between EGF and HRG) or generic expression profiles (highly correlated between EGF and HRG) in the qPCR and microarray data sets. We compared the overlap in these gene lists with those obtained from the CAGE data set. A statistically significant overlap was observed for the microarray data set (two-sample Fisher’s exact test, P-value = 1.38×10^-6^ with OR = 2.24, see Table 2).

**Table 2.** Overlap in stimulus-specific and generic genes between the microarray data set and the CAGE data set.

|  | Microarray-identified Generic Genes | Microarray-identified Stimulus-specific Genes |
| --- | --- | --- |
| CAGE-identified Generic Genes | 133 | 74 |
| CAGE-identified Stimulus-specific Genes | 246 | 307 |

**Table 3.** Overlap in stimulus-specific and generic genes between the qPCR data set and the CAGE data set.

|  | qRT-PCR-identified Generic Genes | qRT-PCR-identified Stimulus-specific Genes |
| --- | --- | --- |
| CAGE-identified Generic Genes | 32 | 24 |
| CAGE-identified Stimulus-specific Genes | 62 | 41 |

For the qPCR data set, the overlap observed was not statistically significant (P-value = 0.738, see Table 3). There were two factors that could have contributed to the lack of significance observed in the overlap of these gene lists. First, the resolution of gene coverage was not the same in the qPCR data set, where only transcription factors were profiled, compared to the CAGE data set which was genome-wide. Second, the time course design was sparser in the qPCR data set where data was collected from six time points during 0 to 6hrs post-exposure to EGF or HRG, whereas for the CAGE data set, there were 16 time points between 0 to 8hrs post-exposure to EGF or HRG. While the time intervals overlap between the CAGE and qPCR data sets, it is possible that with the finer sampling in the CAGE data set, it was easier for the models to distinguish genes with stimulus-specific versus generic expression profiles. Due to the lack of significant overlap between CAGE and qPCR data sets, we chose to consider the comparison of further results between the CAGE and microarray data sets only.

It is worth highlighting that amongst the 32 generic genes that were classified consistently in both CAGE and qPCR data sets, there were several important transcription factors namely EGR1, FOS, SP3, SMAD3, BRIP1. Similarly for the 41 stimulus-specific genes, we saw key regulators such as FOSL2, FOXA1, JUN, JUNB that were classified consistently in CAGE and qPCR data sets. Table 4 lists the generic and stimulus-specific genes that were consistent to both these data sets (sorted alphabetically).

**Table 4.** Genes that were classified consistently between qPCR and CAGE data sets.

| **Generic Genes** | **Stimulus-Specific Genes** |
| --- | --- |
| BCL3, BRIP1, BTG1, BZW1, CITED4, DDX5, EGR1, EIF5B, ELF3, FOS,  GATA3, HEY2, HIST1H2BC, HIST1H2BG, HIST1H2BN, ING1, ING3, KLF7, MAFB, NCOA4, NR4A2, PAX9, PER1, PITX1, RORC, SIAH2, SKIL, SMAD3, SP3, TFAP2C, TFAP4, ZNF467 | ACTB, ARID3B, ATF4, CBX3, CEBPB, CEBPD, CENPB, DEK, DOT1L, EAF1, ELF4, FOSL2, FOXA1, HAX1, HIST1H2BE, IRF1, IRF7, JDP2, JUN, JUNB, KLF16, MAP2K3, MARS, MAZ, MYBBP1A, NFKB2, NFKBIA, OVOL1, POLR1B, PPAN, PXN, RARA, RRS1, SLC2A4RG, TFAP2A, TRIB3, TRIM25, TRIM29, ZFP36, ZNF324, ZNF398 |

*Investigating expression activity of individual generic and stimulus-specific genes in microarray and CAGE data sets.*

We inspected the gene expression profiles of individual generic and stimulus-specific genes in both microarray and CAGE data sets to determine whether similar patterns of expression activity could be observed. Two genes, STRAP and CYTH2 were classified as generic in both CAGE and microarray data sets. The expression profiles for CYTH2 appeared to be quite similar in the microarray data set (Fig. 1B) compared to the CAGE data set (Fig. 2B). For STRAP, the microarray profile shows more divergence between the EGF and HRG time courses (Fig. 1A) than in the CAGE profile (Fig. 1B).

Two genes, FHL2 and FLNA were both classified as stimulus-specific genes in the microarray and CAGE data sets. Although both genes have profiles that diverge between the EGF and HRG time courses, it appears as if the timing of this divergence is somewhat different between the CAGE and microarray data sets. For instance, FHL2 has initial divergence in the microarray profile (Fig. 1C) where this divergence occurs much later in the CAGE profile (Fig. 2C). Similarly, the observation pertains to FLNA (Figs. 1D and 2D). This could be due to the differences in sampling of time points for in CAGE and microarray data sets where the later occurs between 0 to 8hrs, while the microarray data set occurs between 0 and 72hrs. It is highly possible that the later phases of the CAGE profile correspond to the earlier phases of the microarray profile.

In the main paper we had highlighted four stimulus-specific genes that were associated with significant fold changes in the EGF and HRG time courses in the CAGE data. These were PHLDA2, TNFRSF11B, EGR1 and DUSP5. Inspection of the same four genes in the microarray data showed that they also adopted similar patterns of divergent expression (see Fig. 3).

**Fig 1.** Individual gene expression profiles from the microarray data set. A. STRAP and B. CYTH2 are two examples of generic genes. C. FHL2 and D. FLNA are two examples of stimulus-specific genes.


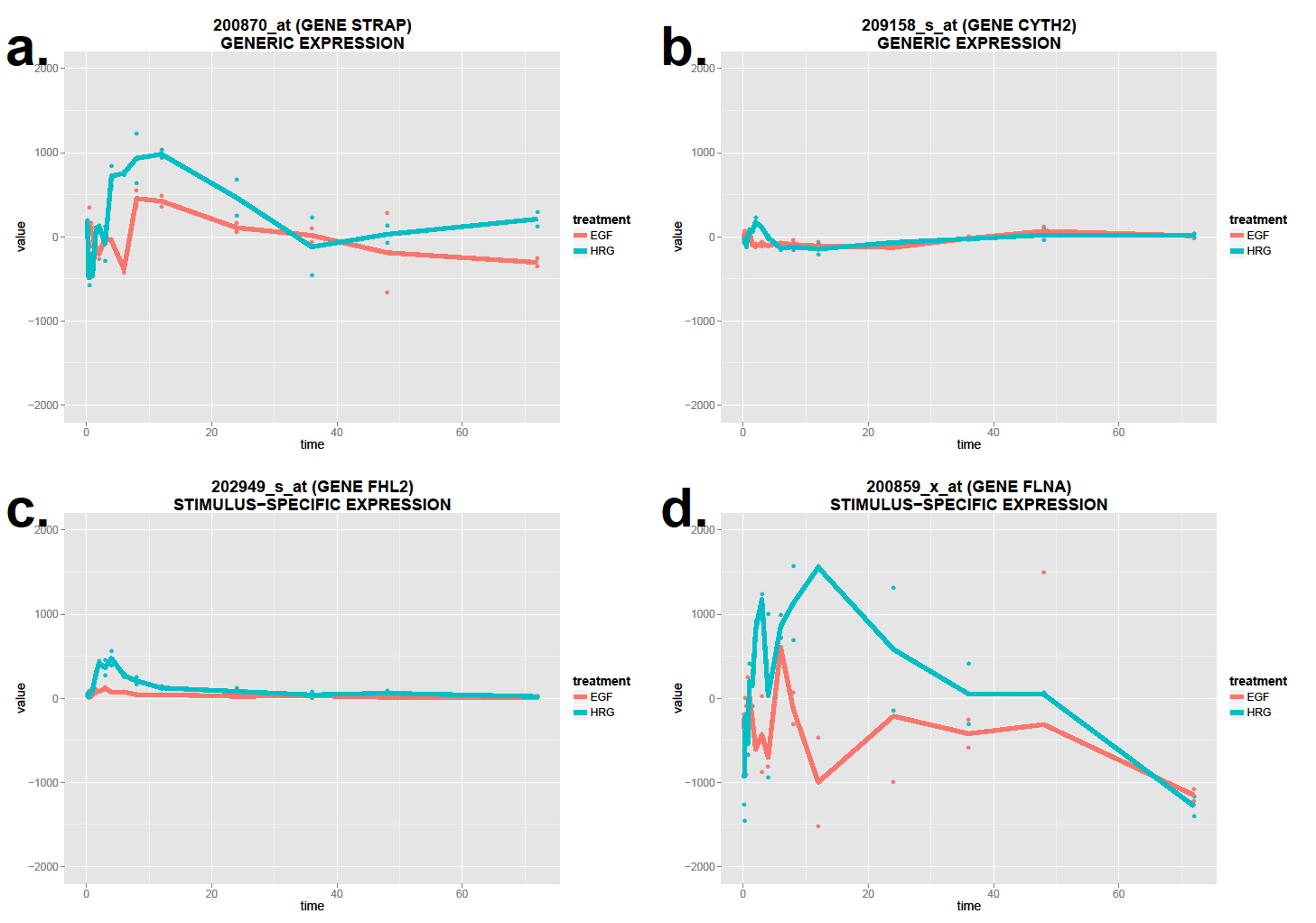


**Fig 2.** Individual gene expression profiles from the CAGE data set. **A.** STRAP and **B.** CYTH2 are two examples of generic genes. **C.** FHL2 and **D.** FLNA are two examples of stimulus-specific genes.


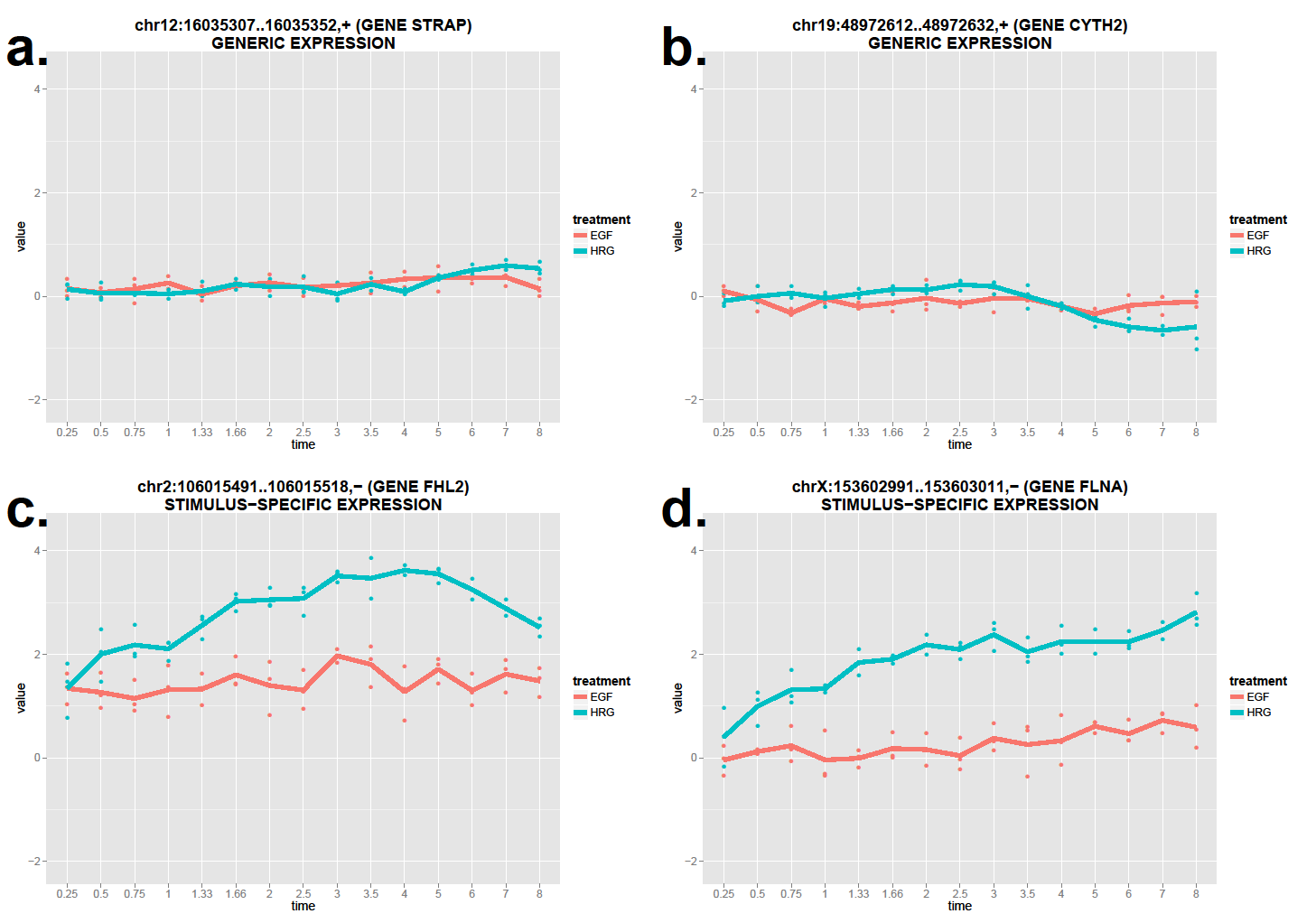


**Fig. 3.** Individual gene expression profiles from the microarray data set for four stimulus-specific genes that had significant changes in the EGF and HRG time courses. **A.** PHLDA2, **B.** TNFRSF11B, **C.** EGR1 and **D.** DUSP5.


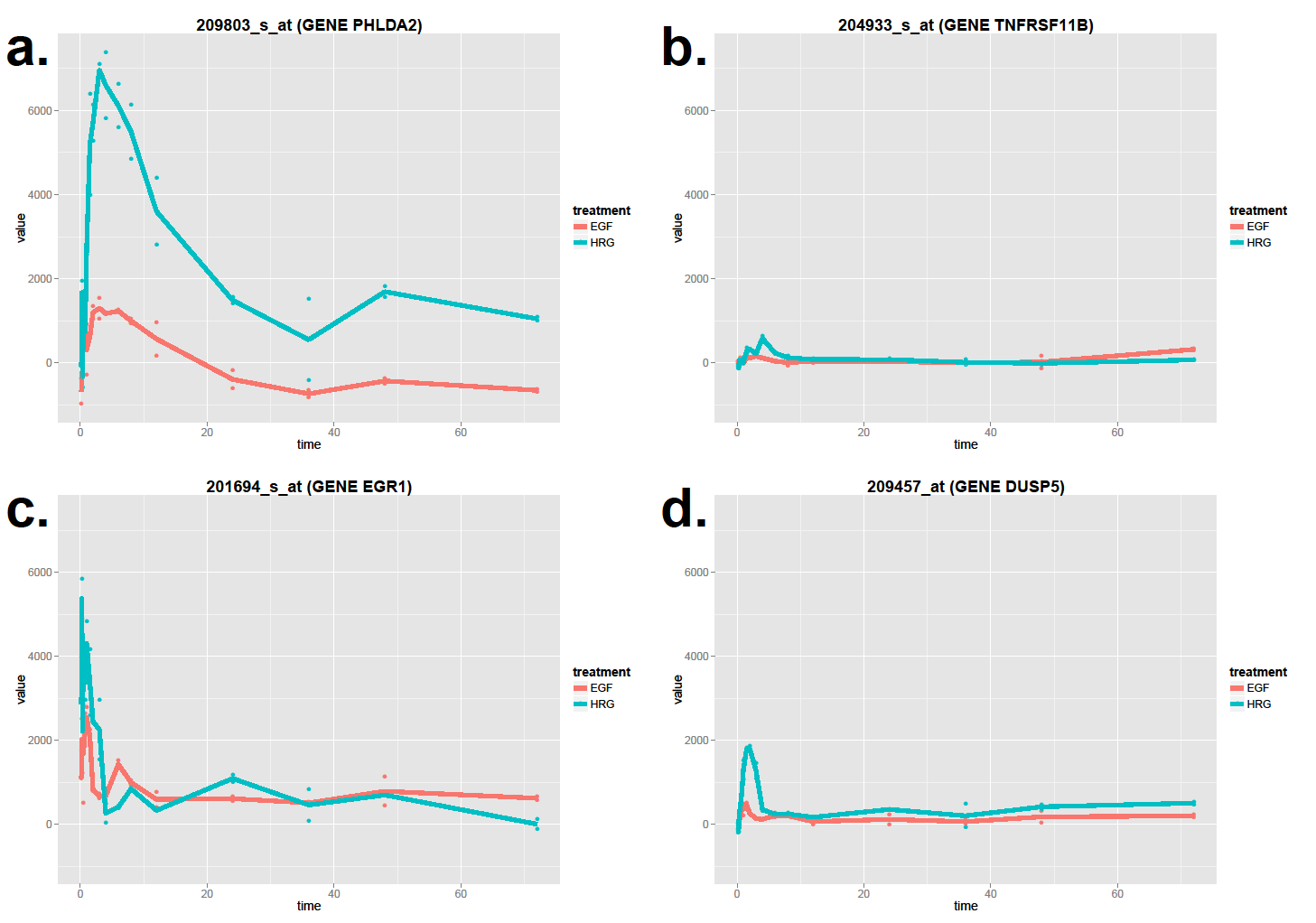


*Validation of enriched pathways in generic and stimulus-specific genes in microarray and CAGE data sets.*

We tested the lists of stimulus-specific and generic genes identified from the microarray data set for enrichment of GO terms and KEGG pathways. We compared the statistically-significant terms with those obtained from the CAGE data set, and identified some terms that were observed in both CAGE and microarray data. For the generic genes, overlapping terms for GO:BP were “negative regulation of transcription, DNA-templated”, “positive regulation of apoptotic genes”; for GO:CC were nucleoplasm, nucleus, cytoplasm, cytosol; and for GO:MF protein binding, transcription coactivator activity (see Table 5).

For the stimulus-specific genes, overlapping terms were for GO:BP “positive regulation of transcription from RNA polymerase II promoter”, “negative regulation of transcription from RNA polymerase II promoter”, “negative regulation of transcription, DNA-templated”; for GO:CC nucleus, nucleolus, focal adhesion; and for GO:MF protein binding. The list of stimulus-specific genes identified from the CAGE data set were enriched for some KEGG terms, and the ones that overlapped with the microarray data were p53 signaling pathway, Shigellosis, MAPK signaling pathway, pathogenic *Escherichia coli* infection, and bladder cancer (see Table 6).

While there are some clear data-specific differences observed in the results obtained from the CAGE and microarray data sets for functional enrichment, overall there is a smaller subset of significant terms were observed in both data sets. Taken together these comparisons suggest that there is some commonality between the results obtained from the CAGE data set and those from the microarray data set.
